# A comprehensive review on the neuroprotective potential of resveratrol in ischemic stroke

**DOI:** 10.1016/j.heliyon.2024.e34121

**Published:** 2024-07-05

**Authors:** Maryam Owjfard, Zahra Rahimian, Farzaneh Karimi, Afshin Borhani-Haghighi, Arashk Mallahzadeh

**Affiliations:** a Clinical Neurology Research Center, Shiraz University of Medical Sciences, Shiraz, Iran; b Behbahan Faculty of Medical Sciences, Behbahan, Iran

**Keywords:** Ischemic stroke, Resveratrol, Neuroprotective, SIRT1, Nrf2

## Abstract

Stroke is the second leading cause of death and the third leading cause of disability worldwide. Globally, 68 % of all strokes are ischemic, with 32 % being hemorrhagic. Ischemic stroke (IS) poses significant challenges globally, necessitating the development of effective therapeutic strategies.

IS is among the deadliest illnesses. Major functions are played by neuroimmunity, inflammation, and oxidative stress in the multiple intricate pathways of IS. Secondary brain damage is specifically caused by the early pro-inflammatory activity that follows cerebral ischemia, which is brought on by excessive activation of local microglia and the infiltration of circulating monocytes and macrophages.

Resveratrol, a natural polyphenol found in grapes and berries, has shown promise as a neuroprotective agent in IS. This review offers a comprehensive overview of resveratrol's neuroprotective role in IS, focusing on its mechanisms of action and therapeutic potential. Resveratrol exerts neuroprotective effects by activating nuclear factor erythroid 2-related factor 2 (NRF2) and sirtuin 1 (SIRT1) pathways. SIRT1 activation by resveratrol triggers the deacetylation and activation of downstream targets like peroxisome proliferator-activated receptor-gamma coactivator 1 alpha (PGC-1α) and forkhead box protein O (FOXO), regulating mitochondrial biogenesis, antioxidant defense, and cellular stress response. Consequently, resveratrol promotes cellular survival and inhibits apoptosis in IS.

Moreover, resveratrol activates the NRF2 pathway, a key mediator of the cellular antioxidant response. Activation of NRF2 through resveratrol enhances the expression of antioxidant enzymes, like heme oxygenase-1 (HO-1) and NAD(P)H quinone oxidoreductase 1 (NQO1), which neutralize reactive oxygen species and mitigate oxidative stress in the ischemic brain. Combined, the activation of SIRT1 and NRF2 pathways contributes to resveratrol's neuroprotective effects by reducing oxidative stress, inflammation, and apoptosis in IS.

Preclinical studies demonstrate that resveratrol improves functional outcomes, reduces infarct size, regulates cerebral blood flow and preserves neuronal integrity. Gaining a comprehensive understanding of these mechanisms holds promise for the development of targeted therapeutic interventions aimed at promoting neuronal survival and facilitating functional recovery in IS patients and to aid future studies in this matter.

## Highlights

• Stroke outcomes are exacerbated due to secondary damage caused by inflammation and oxidative stress.
• Resveratrol exhibits anti-inflammatory, anti-oxidative and anti-apoptotic effects in ischemic stroke.
• Resveratrol applies its neuroprotective effects in ischemic stroke through the SIRT1 and Nrf2 pathways.

## Graphical abstract

**Figure:**
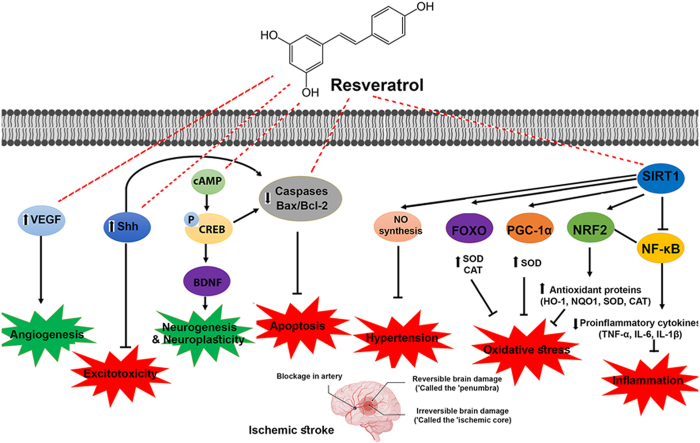

## 1 Introduction

Stroke is the second largest reason for mortality and also the third greatest factor of disabilities worldwide [1]. Depending on the underlying pathology, strokes can be classified as either ischemic or hemorrhagic; worldwide, 32 % are hemorrhagic and 68 % of strokes are ischemic [1,2].

Ischemic stroke (IS) is caused by a disruption of blood flow to the brain. In IS, two different damage regions have been determined: the lesion core, where cells die quickly, and the penumbra (the area around the lesion core), where cells are functionally compromised but may recover and restore function [[3], [4], [5]]. IS is the world's second biggest cause of mortality, with 5.9 million deaths and 102 million disability-adjusted life years lost [5]. IS starts a series of excitotoxicity processes, like ATP depletion, ionic dysregulation, inflammatory processes, oxidative injury, apoptosis, angiogenesis, raised glutamate release, excessive generation of free radicals, apoptosis, and necrosis; all these occurrences ultimately lead to cell death [2,6,7].

Tissue plasminogen activator (tPA) is currently the only FDA-approved treatment for IS [8]. This treatment option comes with a series of limitations such as a narrow therapeutic time window, meaning that not every patient is eligible for treatment [9]. Even in those who are eligible and receive tPA therapy, outcomes depend on the severity of the stroke and may not be significantly improved. This may be due to the reperfusion injury caused by restoring blood flow to the ischemic area which initiates an inflammatory response, generation of free radicals, increased activity in lipases, and endonucleases and therefore worsening of neurological status [10]. These limitations and failure to recover the dying neurons urge us to search for an alternative approach or additive treatments to combine with tPA.

Resveratrol and its anti-inflammatory, anti-apoptotic, and anti-oxidant effects have shown significant health benefits in multiple diseases such as cancer, cardiovascular disease, diabetes and infectious disease [11].

Resveratrol (3,5,4′-trihydroxystilbene) (PubChem CID: 445154) is a naturally occurring phytosterol that resembles estrogen and is mostly present in grapes, peanuts, blueberries, red wine, and other dietary components [12]. Multiple studies have demonstrated that resveratrol presented protective effects in IS, it can mediate blood pressure and lipid profiles which are the main key factors in managing and preventing stroke [[13], [14], [15], [16], [17]]. In this review, we aim to summarize how resveratrol may have a beneficial impact on stroke outcomes and to discuss its therapeutic potential based on previous published literature and laboratory findings.

## 2 Ischemic stroke cascade

When the brain is deprived of the proper blood supply, the ischemic core undergoes infarction and neuronal death immediately. Following the acute phase, multiple processes occur around the ischemic core called the brain penumbra leading to secondary brain injury [18].

As the brain undergoes ischemia, energy stores drop, causing an ionic imbalance and the release of excitotoxic neurotransmitters such as glutamate. The increased release of glutamate and inhibition of its reuptake lead to the overactivation of ionotropic N-Methyl-d-aspartate (NMDA) and α-amino-3- hydroxy-5-methyl-4-isoxazolepropionic acid (AMPA) receptors. Glutamate receptor activation promotes fatal amounts of calcium to enter the cell. This calcium overload leads to the activation of proteases, phospholipases and nucleases which further disrupt membranes and proteins essential for cell function and generate free radicals that are damaging to neurons [[19], [20], [21]]. These events initiate subsequent damage caused by oxidative stress, inflammation, and rupture of the blood brain barrier (BBB) [22,23].

Injured neurons release damage-associated molecular patterns (DAMP) which are responsible for triggering postischemic inflammation [24,25]. DAMPs like interleukin (IL)-33, heat shock proteins (HSP) and adenosine are involved in IS [25,26]. Immune cells detect these danger signals and activate intracellular signaling pathways essential for triggering immune responses [27]. The brain's resident inflammatory cells, microglia, generate pro-inflammatory cytokines like IL-1β, tumor necrosis factor (TNF)-α, and IL-6 in the early phases of IS [28]. The proinflammatory transcription factor kappa B (NF-kB) performs a critical role in cytokine production and the inflammatory response [29]. These cytokines cause endothelial damage, resulting in increased BBB permeability [30]. The microvascular damage and BBB breakdown exacerbate the inflammation in the ischemic region by increasing the permeability and migration of peripheral blood cells to the brain [30,31]. This innate immune system further provokes BBB dysfunction by releasing even more cytokines, reactive oxygen species (ROS), and the chemokine matrix metalloproteinase (MMP)-9, creating cerebral edema and expanding the infarct region [32,33]. These processes, which are all comprised of inflammation, oxidation, autophagy, necrosis, and apoptosis lead to neuronal death [34]. Mediating the immune response has shown neuroprotective effects against IS and therefore improving stroke outcomes [35].

### 2.1 Role of microglia in ischemic stroke

IS is complicated and involves several stages,including BBB disruption, oxidative stress, neuroinflammation, neuroexcitotoxicity, and microglial activation [36]. After ischemia, many factors such as necrotic cells, ROS, and damaged tissues cause inflammation, which in turn triggers the activation of inflammatory cells such as microglia [37]. The CNS's resident macrophages, known as microglia, which make up about 20 % of the overall glial population, are the first cells to respond to IS [38]. Although microglia activation is deleterious in IS, it is essential for boosting neurogenesis, reducing neuronal death, and enhancing functional recovery following cerebral ischemia [36]. Neuroinflammation is criticalin the development and progression of ischemic stroke [39]. Microglial-mediated neuroinflammation is not an independent process and has intricate interactions with other pathological mechanisms like oxidative/nitrative stress, excitotoxicity, necrosis, apoptosis, pyroptosis, autophagy, and adaptive immune reactions [40].

Microglia, when activated, variously express numerous channel proteins, receptors, and enzymes that are associated with boosting or preventing inflammatory processes, resulting in them being an area of intervention for ischemic stroke [40].

Microglia are classified into three kinds: M0 (surveillance), M1 (pro-inflammatory), and M2. (anti-inflammatory) [7]. M0 is mainly accountable for surveillance and exhibits poor phagocytosis and inactivity [41,42]. M1 phenotypic polarization results in the production of pro-inflammatory compounds that impair CNS recovery, while M2 phenotypic polarization results in the release of cytokines that are anti-inflammatory and promote tissue repair and regeneration [43]. M1 microglia release pro-inflammatory molecules like IL-1, IL-6, IL-1β, TNF-α, and IFN-γ which have cytotoxic effects on neurons by increasing the production of inducible nitric oxide synthase (NOs), which leads to the death of neurons [36,44]. These cytokines andMMPs exert essential functions in BBB destruction in IS. They create a rise in adhesion molecules and inflammatory blood cells, particularly neutrophils, which penetrate via the compromised BBB [45]. Compared to M1 microglia, M2 microglia have a greater ability to initiate phagocytosis of dying cells, which can limit the ensuing inflammation and facilitate tissue regeneration [46,47].

### 2.2 Role of astrocytes in ischemic stroke

Astrocytes are the most prevalent kind of glial cell in the brain, comprising approximately 40 % of all brain cells. Astrocytes are classified into three distinct groups based on their shape and spatial organisation:, protoplasmic astrocytes in the grey matter, radial astrocytes that surround the ventricle, and fibrous astrocytes located in the white matter [48]. Astrocytes become active quickly and display two distinct functional phenotypes: the neurotoxic type A1 astrocytes, mostly triggered by inflammation and the neuroprotective A2 type reactive astrocytes, triggered by ischemia.

Astrocytes undergo major alterations in their shape, activity, and molecular expression profile during an IS [49]. Astrocytes become activated during minutes of brain ischemia due to cytokines generated by injured neurons and glial cells in the penumbra and core of the infarct. This is also known as reactive astrogliosis, and it is marked by cell proliferation, hypertrophy, and elevated expression of the glial fibrillary acidic protein (GFAP). It also alters the expression of several molecules that affect cell structure, intracellular signal transmission, gene transcription, energy metabolism, and membrane transport proteins. In addition to supporting structure and metabolism, they can protect the BBB, control blood vessel tone in reply to neuronal activity, remove excess neurotransmitters (glutamate homeostasis), balance oxidative stress, and encourage the development and maintenance of synapses [[50], [51], [52], [53]]. Astrocytes are involved in a variety of ischemia signal-induced procedures, like excitotoxicity, oxidative stress, metabolic dysregulation, edema production, scar-border development, neuroinflammation and finally apoptosis and necrosis of neurons [53]. Astrocytes may be activated by inflammatory substances generated by microglia, including transforming growth factor-alpha (TGF-α), IL-6, leukemia inhibitory factor (LIF), and TNF-α. In addition, dying neurons and endothelial cells contribute to astrocyte activation. They mainly generate cytokines to control the activation and proliferation of astrocytes [48]. Numerous inflammatory compounds, including TNF-α, IL-1α, and IFN-γ, along with free radicals, like NO, peroxynitrite, and superoxide dismutase are generated and released via reactive astrocytes (RA). These agents either indirectly or directly result in neuroinflammation, which in turn causes neuronal apoptosis and necrotic death. Moreover, RAs release cytokines that prevent inflammation from happening [[54], [55], [56]]. The NF-κB signalling pathway promotes the formation of inflammatory-neuronal astrocytes. These astrocytes cause neuronal apoptosis through reregulating complement cascade genes, releasing inflammatory cytokines, and decreasing the production of SPARCL1, GPCG4/6, and ThBS1/2 with neurotrophic operation [[57], [58], [59]]. The JAK2/STAT3 signalling pathway-mediated neuroprotective kind astrocytes up-regulate several neurotrophic factors and support neuronal survival and development, indicating that this kind of astrocyte might serve a beneficialrepair role [60].

## 3 Resveratrol: bioavailability and therapeutic effects

Resveratrol (3,5,4′-trihydroxystilbene) (PubChem CID: 445154) is a naturally occurring phytosterol that resembles estrogen and is mostly present in grapes, peanuts, blueberries, red wine, and other dietary components [12]. Resveratrol has a quick rate of absorption and is absorbed in substantial quantities by enterocytes [61,62]. However, plasma levels of resveratrol are often low owing to high levels of intestine and liver metabolism [62]**.** When resveratrol reaches the gastrointestinal system, it undergoes quick and substantial biotransformation before being distributed to different organs, where it becomes accessible and active [63]. Absorbed resveratrol in intestinal enterocyte cells undergoes sulfation and glucuronidation processes [64]. Resveratrol that has been conjugated leaves the cell through the transporters in the apical and basolateral membranes, while a tiny amount of resveratrol that has not been conjugated leaves the enterocyte through the basolateral membrane [63]. Resveratrol and conjugated metabolites pass through the small intestine's apical membrane and into the large intestine. Here they are metabolized by gut bacteria to produce dihydroxytrans-stilbene, lunularin and dihydroresveratrol [65]. Resveratrol and metabolites that leave the enterocyte enter the portal circulation and the liver, and thus undergo further conjugation [66]. Furthermore, conjugated resveratrol and metabolites pass through enterohepatic circulation, exiting the liver to be reabsorbed in the intestine following hydrolysis and then reaching the portal circulation and returning to the liver for additional metabolism [64]. Resveratrol and its metabolites reach the systemic circulation via the liver and are transported by binding to blood proteins like lipoproteins, hemoglobin, and albumin [67]. The kidneys are involved in the metabolism of resveratrol as well, which results in the excretion of polarised resveratrol metabolites [64]. Resveratrol not only has a quick metabolism, but it also is excreted quickly. A study on rats has shown that 49–60 % of the resveratrol is eliminated in the urine, from the remaining 77–80 % being absorbed in the intestine [61]. Thus, 75 % of the total amount of resveratrol that is ingested is excreted [68]. The residual quantity of resveratrol undergoes metabolism, with the maximum reported concentration of free resveratrol being 1.7–1.9 % [69]. The levels of resveratrol peak 60 min following ingestion. Another study found that within 6 h, there was a further rise in resveratrol levels. This increase can be attributed to intestinal recirculation of metabolites. In the intestines, these metabolites are reabsorbed after being hydrolyzed into their free form [69].

Resveratrol (3,4,5 -trihydroxytrans-stilbene) is a polyphenolic molecule from the stilbene class that occurs in two isomers: cis and trans [70]. Stilbenes, a type of secondary metabolite, eliminates free radicals and protects against chronic illnesses like diabetes, cancer, heart disease, and arteriosclerosis [71]. They also help with aging [72]. However, resveratrol may provide additional health advantages, since various studies have shown that it has antioxidant, anti-inflammatory, neuroprotective, and chemotherapy-protective properties [73].

Resveratrol is commonly found in red grapes (the richest source), cocoa, peanuts, wine, grape juice, berries of *Vaccinium* species like blueberries, cranberries and bilberries [74]. It was found that resveratrol can lower blood pressure in hypertensive rats, providing a novel treatment option for cardiovascular disease [14]. This substance modulates cellular immunity [11]. In addition, resveratrol may protect against neurological illnesses including Parkinson's and Alzheimer's [75]. Recently, researchers studied the therapeutic potential of resveratrol in hemostatic diseases related with COVID-19. This compound's anti-clotting and anti-inflammatory activities have been proven to have a crucial impact in lowering COVID-19 mortality [76]. It is also helpful in various areas, including mitochondrial malfunction, oxidative stress, angiogenesis, apoptosis and inflammation. It inhibits platelet aggregation and has cardioprotective effects [77].

## 4 Effect of resveratrol on astrocytes and microglia

Resveratrol treatment has shown to improve neuronal dysfunction, infarct volume, and neuronal morphological alterations in MCAO animals. Meanwhile, pro-inflammatory microglia activation and inflammatory factor productions were suppressed. CD147 and MMP-9 levels were elevated in primary microglia. Resveratrol inhibited the CD147/MMP-9 axis in OGD/R microglia. All leukocytes, platelets, and endothelial cells contain the transmembrane glycoprotein CD147, which has been demonstrated to be a key mediator of inflammation and immune responses [78].

Activated Smo can enhance neurological performance by controlling oxidative stress, inflammation, apoptosis, neurogenesis, oligodendrogenesis, and axonal remodeling. More investigations have shown that resveratrol can activate Smo. However, it is still unclear whether resveratrol suppresses the activity of microglial cells with Smo. Studies have found that the Smo receptor could be a therapeutic target of resveratrol, assisting to decrease microglial activity in the acute phase of stroke [79]. Resveratrol has a neuroprotective impact versus IS, which is partly due to its stimulation of JAK2/STAT3 and PI3K/AKT/mTOR axis. Resveratrol may indirectly stimulate the PI3K/AKT/mTOR axis via stimulating JAK2/STAT3 [80].

Resveratrol boosts anti-inflammatory and diminishes inflammatory cytokines via altering microglial signalling pathways in microglia like: AMP-activated protein kinase (5′ adenosine monophosphate-activated protein kinase, AMPK), SIRT1 (sirtuin 1) and SOCS1 (suppressor of cytokine signaling). Furthermore, through miR-155 overexpressing in microglia, resveratrol promotes M2 phenotype polarization [81].

The expressions of iNOS and NF-***κ***B p65 subunits in microglial cells were elevated after a 24-h exposure to LPS/IFN***γ***, and the releases of TNF***α*** and IL-1***β*** were also enhanced. Resveratrol decreased the expressions of iNOS and NF-***κ***B p65 subunits as well as the releases of proinflammatory cytokines [82].

Resveratrol also increases AMPK and inhibits GSK-3β (glycogen synthase kinase 3 beta) activity in astrocytes, which release energy, makes ATP available to neurons and reduces ROS. . Furthermore, oligodendrocyte survival is boosted by resveratrol, which may help to preserve brain homeostasis following a stroke [81]. Based on these findings, resveratrol may be regarded as a novel therapeutic option for improving the symptoms of stroke. It can influence neuronal function and also significantly lower neurotoxicity by changing glial function and signaling.

## 5 Neuroprotective roles of resveratrol

**Fig. 1:**
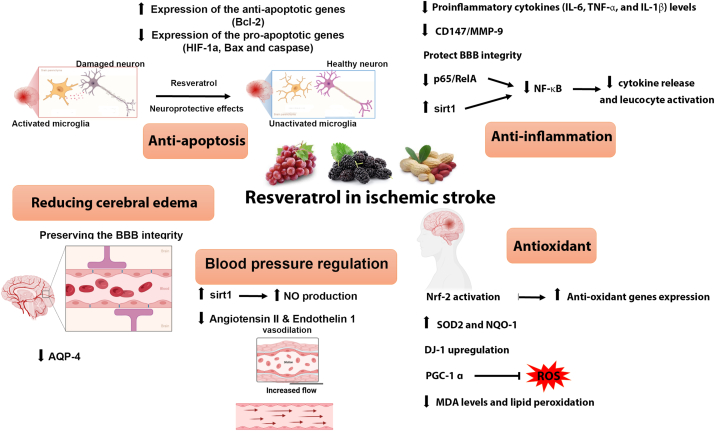
Neuroprotective role of resveratrol in IS. Following cerebral ischemia, the secondary damage continues to affect the brain and injure neurons due to immune cell infiltration, oxidative stress and BBB disruption. Resveratrol holds the potential to inhibit inflammatory cytokine release and preserve the BBB integrity through the activation of the SIRT1 and Nrf2 pathway. Resveratrol upregulates the expression of anti-oxidant genes including Superoxide dismutase (SOD)2 and NAD(P)H quinone oxidoreductase 1 (NQO-1). Resveratrol also stimulates the expression of the Bcl-2 anti-apoptotic gene while suppressing the expression of apoptotic genes like Bax, caspase and Hypoxia-inducible factor 1-alpha (HIF-1a). Resveratrol is capable of reducing cerebral edema by preserving the BBB integrity and reducing the expression of aquaporin (AQP)-4. The SIRT1 pathway leads to increased nitric oxide (NO) production. Increased NO along with the reduction in angiotensin II and endothelin 1 contribute to resveratrol effects on regulating blood pressure and cerebral blood flow.

Resveratrol has neuroprotective benefits in both IS and intracerebral hemorrhage. Atherosclerosis is regarded as a risk factor for IS. In this regard, resveratrol may suppress platelet activation and aggregation caused by collagen, adenosine diphosphate, and thrombin. The postulated mechanism encompasses the suppression of tissue factor gene expression or the manufacture of prothrombotic agents [83]. Intracranial hypertension and cerebral edema are frequent consequences of a cerebral infarction and can lead to death. A variety of therapies that effectively combat cerebral edema have been created in animal experiments, a number of which have been evaluated in clinical trials; among them, resveratrol has shown edema reducing effects. Although resveratrol is a highly hydrophobic molecule, it is exceedingly difficult to penetrate a membrane like the BBB. However, an alternate administration is through the nasal cavity in the olfactory area, which results in a more pleasant route for the patient. Resveratrol has multiple approaches of action associated to its effects on stroke, since the molecule interacts with a broad range of enzymes and receptors, enhancing stress resistance and lowering apoptosis [[84], [85], [86]]. In addition to apoptosis, several other pathophysiologic processes like inflammation, oxidative stress, and ionic imbalance also work in concert to cause brain damage and neuronal death following a stroke [87]. Resveratrol is being widely investigated as a potential therapeutic agent in IS due to its anti-inflammatory, antioxidant, anti-tumorigenic,anti-aggregation, and edema reducing properties [88] (Fig. 1).

### 5.1 Anti-inflammation

Resveratrol's anti-inflammatory effects have been demonstrated in many studies. One of the major roles of resveratrol in attenuating inflammation is through the activation of SIRT1 [89,90]. Resveratrol is known as the most effective up regulator of SIRT1 [91]. SIRT1 is a deacetylase that plays a key role in maintaining immune tolerance and regulating T cell function [92,93]. Evidence-based studies have implicated the effects of SIRT1 activation in reducing inflammation and alleviating immune response [94,95]. SIRT1 is widely expressed in the CNS and therefore, overactivation of SIRT1 can lead to regulating postischemic neuroinflammation [96]. One of resveratrol's substrates is p65/RelA which is a member of the NF-kB transcription factor that significantly increases cytokine release and leukocyte activation [97,98]. Cytokines released due to ischemic conditions disrupt the BBB and increase its permeability allowing monocyte, neutrophil, and leukocyte infiltration which aggravates inflammation in the ischemic area [28]. Resveratrol binds to SIRT1 and modulates its structure, facilitating its binding to substrates [99]. This enhances RelA acetylation which results in inhibition of the NF-kB dependent cytokine production like, IL-1β, IL-6, and MMP-9 [100,101]. Hence, resveratrol exhibits the potential to protect the BBB against leakage and immune cell infiltration and therefore, limit neuron loss [102]. A study performed on cortical mixed glial cells exposed to hypoxia/hypoglycemia demonstrated that IL-6 gene expression and excretion was reduced following resveratrol treatment in a dose dependent manner [103]. In a mouse model of MCAO, resveratrol administration exhibited dose-dependent reductions in IL-1β, IL-6, TNF-α, and ROS production in the ischemic cortex, and ultimately decreased infarct volumes as compared to the control group [104,105]. Shin et al. evaluated IL-1β and TNF-ɑ mRNA and protein levels in an MCAO model. They found that the mRNA and protein levels of these two cytokines were markedly diminished in the resveratrol-treated group [105]. Another study demonstrated that myeloperoxidase activity, which indicates neutrophil infiltration, was reduced in MCAO rats treated with resveratrol [106]. Resveratrol was found to lower cytokine levels in both the central nervous system and the peripheral blood, suggesting that this treatment option may help preserve the integrity and structure of the blood-brain barrier [107]. Resveratrol can also suppress the CD147/MMP-9 pathway, which in turn inhibits the production of cytokines from microglia in the ischemic brain, according to a recent animal research [78]. IS etiology appears to be significantly influenced by CD147 and MMP-9 [108]. The experiment's findings demonstrated that 24 h following brain ischemia, MPO activity was noticeably increased. However, this increase shown a substantial decrease following resveratrol medication [109]. Resveratrol dramatically lowered the amounts of cerebral infarcts, neuronal damage, MPO activity, and evans blue (EB) content in addition to neurological impairment scores. TLR4, NF-κB p65, COX-2, MMP-9, TNF-α, and IL-1β all had greater levels of expression after cerebral ischemia, whereas resveratrol decreased these amounts [109].

### 5.2 Anti-oxidative

Oxidative stress is when cells are exposed to molecular oxygen or its derivatives, particularly ROS, and the cells fail to protect themselves against cellular damage [110]. ROS production and oxidative stress, damage neurons in ischemic situations and play a key role in ischemic reperfusion injury [111]. Resveratrol applies its anti-oxidant effects through diverse mechanisms and pathways that activate anti-oxidant enzymes [112].

One of the major pathways that resveratrol activates to fight against oxidative stress is the nuclear factor-erythroid 2-related factor 2 (Nrf2) pathway. The Nrf2 transcription factor is responsible for anti-oxidant gene regulation and protects cells from oxidative damage [113]. During normal conditions, Nrf2 stays in its inactive form by binding to the keap1 protein [114]. During stressful situations and ROS accumulation, the keap1 protein dissociates from Nrf2 and leads to anti-oxidant gene expression [115,116]. Resveratrol can lead to the dissociation of keap1 from Nrf2 and increase its active form [117]. When Nrf2 is dissociated from keap1, it translocate into the nucleus and heterodimerizes with small Maf proteins, which then bind to the cell's DNA and lead to the transcription of Nrf2 target genes including heme oxygenase-1 (HO-1) [118]. HO-1 can reduce the overproduction of inflammatory cytokines (e.g., TNF-ɑ, IL-6) by inhibiting the NF-kB signaling pathway [119]. The enzyme HO-1 breaks down free heme to produce carbon monoxide (CO), free iron, and biliverdin, which is quickly converted to bilirubin via biliverdin reductase [120]. Unconjugated bilirubin, biliverdin, and CO block NADPH oxidase complexes, which are significant contributors to excitotoxicity [[121], [122], [123]]. HO-1 is a Nrf2-regulated gene with significant antioxidant, anti-inflammatory, antiapoptotic, and antiproliferative properties [124]. Once NADPH oxidase is hyperactive, the ensuing oxidant generation stimulates Nrf2 and thus promotes HO-1, which produces bilirubin, this inhibits NADPH oxidase activity via feedback. Therefore, HO-1 regulates cellular oxidative stress.[125]. Furthermore, when iron free ions are exposed to oxidants like superoxide and hydrogen peroxide, they can form very reactive and deadly oxidant hydroxyl radicals, which can cause neurotoxicity [126]. In addition to biliverdin and bilirubin, CO generated by HO-1 induction inhibits the NADPH oxidase [127]. Low amounts of CO can stimulate the enzyme guanylate cyclase, mimicking the physiological action of NO. The product of this enzyme's activity, cyclic GMP (cGMP), has the ability to activate protein kinase G (PKG), that in turn causes neurotropic activity in neurons through AKT kinase activation. Therefore, therapies which raise cGMP concentrations in neurons may be effective in counteracting excitotoxicity [127]. Since NO also aids in the generation of peroxynitrite, enhancing neuronal generation of NO isn't a suitable way to achieve this.

The expression levels of HO-1 in ischemic cerebrovascular disease patients are significantly increased and positively correlated with the severity of the disease [[128], [129], [130], [131]]. Post-ischemic upregulation of HO-1 appears to be part of the response mechanisms of the brain to reduce neuronal damage [128].

Findings from previous studies suggest that Nrf2 activation can significantly reduce brain injury following IS and lead to better outcomes [132]. A study on the primary Culture of Rat Cortical Neurons indicated that resveratrol significantly reduces brain injury induced via oxygen/glucose deprivation/reoxygenation (OGD/R) in the rat cerebral cortex by upregulating Nrf2 [133]. They used Western blot analysis to show that levels of anti-oxidant molecules such as superoxide dismutase 2 (SOD2) protein and NAD(P)H quinone oxidoreductase-1 (NQO-1) were increased in rat cultures treated with resveratrol compared to the normal group.

Abdel-Aleem, Ghada A. et al. demonstrated that resveratrol can protect the brain against ischemia-reperfusion injury by regulating the DJ-1 protein [134]. This protein has a key role in anti-oxidation and cell survival, but its function is disrupted during ischemia-reperfusion injury and fails to protect neurons [117]. They found that resveratrol reduced oxidized forms of DJ-1 levels, resulting in increased survival in rats with brain damage.

Previous studies have shown that resveratrol activates the PPAR -γ coactivator 1α (PGC-1 α), which has free radical scavenging properties [113,135]. PGC-1α has the potential to regulate SOD2, glutathione peroxidase 1 and thioredoxin, and therefore, alleviate oxidative stress [136]. PGC-1ɑ regulates mitochondrial anti-oxidant mechanisms and prevents ROS accumulation [137]. PGC-1ɑ can be regulated by two resveratrol target pathways [138], adenosine-monophosphate-activated protein kinase (AMPK)-mediated phosphorylation and SIRT1-mediated deacetylation, both of which are involved in increasing PGC-1ɑ activity [139,140]. Animal studies have shown that resveratrol enhances PGC-1ɑ expression in MCAO mouse models [91]. Also, the forkhead box protein O (FOXO) pathway, which is activated through the SIRT1 pathway, plays a key role in mediating cellular oxidative stress [141].

Malondialdehyde (MDA) is a commonly used biomarker for detecting oxidative stress in cells undergoing injury [142]. MDA can react with cell structures such as phospholipids and nucleic acids, which can lead to immune system dysfunction [143]. Studies have shown that serum MDA levels are increased in IS patients [144,145]. Resveratrol administration has been shown to reduce MDA levels and lipid peroxidation in the mouse brain [146].

### 5.3 Anti-apoptosis

Apoptosis, programmed cell death, plays a crucial role in various pathological processes, such as stroke [147]. It may take several hours or days for apoptosis to take place in the brain penumbra following an IS [148]. Due to ionic imbalance and calcium overload in IS, neurons may undergo apoptosis, which leads to a significantly greater amount of cell death [149]. Resveratrol has shown pro-apoptotic properties in cancer [150] while it possesses an anti-apoptotic property in acute CNS insults [138]. Resveratrol induces its anti-apoptotic effects through multiple pathways, including the phosphatidylinositol 3-kinase (PI3K)/protein kinase B (Akt) pathway and the extracellular signal-regulated kinase (ERK) pathway. Activation of these pathways can promote cell survival and inhibit apoptotic signaling [80]. Resveratrol holds the potential to upregulate the Bcl-2 family proteins, which play a key role in apoptosis suppression while downregulating the expression of pro-apoptotic proteins such as Bax [151]. Caspases are key mediators of apoptosis. Resveratrol has been shown to inhibit caspase activation, particularly caspase 3, which is responsible for carrying out the apoptosis process and therefore leading to cell survival [152]. Resveratrol can prevent mitochondrial membrane depolarization, preserve adenosine triphosphate (ATP) production, and inhibit the release of cytochrome *c*. In addition, mitochondrial lipid peroxidation (LPO), protein carbonyl, and intracellular hydrogen peroxide (H2O2) content were significantly reduced in the resveratrol treatment group, while the expression of HSP70 and metallothionein were restored [153]. Metallothionein and HSP70 are stress proteins involved in protection against oxidative damage [154]. Oxidative damage following a stroke can induce apoptosis in cells [155]. Resveratrol's anti oxidative effects, discussed earlier, also contribute to reducing the risk of apoptosis. Wang et al. demonstrated that resveratrol reduced neurocyte apoptosis, as evidenced by an enhanced Bcl-2/Bax ratio, and lowered the levels of apoptotic cells (TUNEL positive cells) [156]. In female rats treated with resveratrol for acute global cerebral infarction, Bcl-2 levels were significantly elevated, but p53 levels as an apoptotic marker were significantly decreased [157].

### 5.4 Edema reducing effects

Cerebral edema can aggravate stroke outcomes. This lethal condition occurs within 48 h post IS and peaks 3–5 days after stroke occurrence [158]. Ischemic conditions cause tissue necrosis and basal membrane breakdown, leading to BBB impairment [159,160]. This allows serum proteins to improperly shift from the blood to the brain and lead to a type of vasogenic edema which significantly affects the degree of neurologic deficit [158]. A study performed on rats demonstrated that resveratrol inhibited brain edema in IS. Resveratrol also decreased the levels of plasma membrane channels responsible for water hemostasis called Aquaporin (AQP)-4 [161]. Resveratrol's edema reducing effects are due to its impacts on preserving BBB integrity and its ability to lower AQP-4 levels.

### 5.5 Effects of resveratrol on blood pressure and cerebral blood flow

The mechanisms underlying the blood pressure-lowering effects of resveratrol are not fully understood but may involve multiple pathways. Resveratrol has been found to enhance NO production, a potent vasodilator, which can lead to relaxation and widening of blood vessels. By promoting NO-mediated vasodilation, resveratrol may help improve cerebral blood flow (CBF) [162]. In addition, resveratrol has been shown to inhibit angiotensin II, a hormone that can constrict blood vessels and lead to high blood pressure [163] The ability of resveratrol to modulate these pathways may contribute to its beneficial effects on blood pressure regulation. Moreover, resveratrol has been reported to possess antioxidant and anti-inflammatory properties as discussed earlier, which could also play a role in blood pressure regulation. Oxidative stress and chronic inflammation are known to contribute to endothelial dysfunction and vascular damage, both of which are associated with hypertension [13,164]. By reducing oxidative stress and inflammation, resveratrol may help improve endothelial function and maintain healthy blood pressure levels and therefore, improve CBF [165,166]. It has been suggested that resveratrol can influence the release and activity of vasoactive substances, such as endothelin-1 and prostaglandins, which can modulate the diameter of blood vessels in the brain [167,168]. By modulating these vasoactive substances, resveratrol may regulate cerebral microcirculation and impact CBF.

## 6 Animal experiments with resveratrol in IS

Multiple studies have experimented with the effects of resveratrol on various stroke outcomes. *Shin* et al. showed that 5 mg/kg intravenous (IV) resveratrol reduced infarction volume by 36 % in an MCAO mouse model. The neuroprotective effects are due to inflammatory suppression as well as ROS inhibition in the ischemic cortex [105]. Another study by the same authors confirmed their previous findings when resveratrol reduced total infarct volume by 45 % [91]. Their results also showed that resveratrol activates the transcription factor cAMP-response-element binding protein (CREB). CREB enhances cortical circuit plasticity and is involved in forming new connections which lead to recovery from stroke motor deficits [169]. Rats pre-treated with nanostructured lipid carriers containing resveratrol (NR) for 10 days before MCAO showed improved behavioral tests and decreased infarct volume in a dose-dependent manner. The increased activities of caspases 3 and 9 as well as cytokines (IL-1, 6, and TNF- α) in the MCAO group were considerably prevented by 500 μg of NR administration. This study indicates that resveratrol holds the potential to improve stroke outcomes before ischemia as a pre-treatment strategy [104]. Resveratrol treatment after a single or repeated mild stroke has been shown to decrease brain injury, prevent cerebral edema, protect endothelial cells and preserve BBB function [170]. *Faggi* et al. found that resveratrol treatment significantly reduced the infarct volume in mice with MCAO. They did minimize the infarct volume when administered at higher doses (resveratrol 6.8 mg/kg). According to the findings of this study, resveratrol may be an effective ready-to-use treatment for treating post-ischemic brain injury [171]. Another study found that *trans*-resveratrol treatment significantly reduced infarct volume and prevented motor impairment, increased glutathione, and decreased MDA levels compared to the control group, suggesting that *trans*-resveratrol could be one of the medications used for minimizing neurologic deficits due to stroke [172]. In the delayed phase after stroke, resveratrol treatment via gavage has been shown to be useful for reducing infarct volume and improving neurological impairments. The increased MMP-2 and vascular endothelial growth factor levels may play a role in the neuroprotective effect of resveratrol treatment by causing angiogenesis [16]. Another research article emphasized the importance of resveratrol in the treatment of ischemic stroke due to its capacity to preserve the structure and function of ischemic neurovascular units. Resveratrol significantly reduced neurological deficits as evaluated by several scoring techniques, brain infarct volume, and brain edema [153]. The TRPC6-MEK-CREB and TRPC6-CaMKIV-CREB pathways were identified by *Lin* et al. as potential mechanisms by which resveratrol protects neurons from ischemia injury. After cerebral ischemia/reperfusion injury, pretreatment with resveratrol for 7 days significantly reduced neurological deficits, and the infarct volumes were associated with increased TRPC6 and *p*-CREB activity [173]. Resveratrol treatment significantly decreased infarct sizes, improved neurobehavioral deficits, and prevented neuronal cell death in both in vivo and *in vitro* models of recurrent ischemic stroke. Resveratrol treatment significantly enhanced the intracellular NAD^+^/NADH ratio, as well as AMPK and SIRT1 activities and, decreased energy ATP requirements during ischemia, consequently suggesting the neuroprotective properties of resveratrol [174]. Fang et al. found that resveratrol was able to decrease the extent of cerebral infarction, brain water content, neuronal apoptosis, myeloperoxidase levels (which are expressed in immune cells), and cerebral TNF-α production in a rat model of focal cerebral ischemic reperfusion injury [175]. Pretreatment with 50 mg/kg resveratrol for 7 days was shown to protect against cerebral ischemia/reperfusion injury via enhanced anti-apoptosis, anti-inflammation, and autophagy activation. Resveratrol significantly lowered the neurologic deficit, cerebral infarct volume, the levels of caspase-3 and IL-1β, but also significantly increased the ratios of Bcl-2/Bax [176]. When given 6 days before MCAO, resveratrol significantly decreased neurological deficits and infarct volume. The authors hypothesized that resveratrol's neuroprotective effects may be via inhibiting phosphodiesterase, and subsequently activating the AMPK/SIRT1 signaling pathway, and lowering ATP energy consumption in neurons during ischemia [177]. Intra-carotid artery administration of resveratrol polymeric nanoparticles showed significant protection against cerebral ischemia/reperfusion injuries, as evidenced by improved neurological functions, decreased infarction volumes, preservation of the BBB, prevention of brain edema, attenuation of oxidative stress, inhibition of neuronal apoptosis, and promotion of neurogenesis through increased expression of brain-derived neurotrophic factor and Bcl-2/Bax ratio [178]. The brain-derived neurotrophic factor is involved in increasing neuroplasticity and stroke recovery. Treatment with resveratrol after MCAO improved neurobehavioral deficit, brain water content, infraction, and cerebral cortex histological alterations. In addition, resveratrol prevented the declines in phospho-Akt and phospho-GSK-3 protein levels that were induced by MCAO injury [179]. Different neurological scoring tests, including cylinder test, spontaneous motility, righting reflex, horizontal bar test, forelimb flexion, actophotometer, rotarod, Randall Sellito and Von Frey were all improved by chronic doses of resveratrol (20 mg/kg) for 21 days. Resveratrol also decreased the immobility time forced swim test and the Morris water maze memory deficit [180]. When resveratrol was administered during both gestation and lactation (2 weeks prior to the hypoxic-ischemic in pups), the greatest recoveries were observed. The authors hypothesize that resveratrol affects brain metabolism, especially the astrocyte-neuron lactate shuttle, which contributes to neuroprotective properties [181]. Pretreatment with resveratrol (20 or 40 mg/kg) significantly lowered the cerebral edema, infarct volume, lipid peroxidation products, and inflammatory markers, including IL-1β, IL-6, TNF-α, NF-κB p65 subunit and significantly increased the antioxidant capacity by enhancing the activities of glutathione peroxidase, catalase, SOD; and signified an increase in HO-1, and Nrf2 [182]. Intraperitoneal administration of resveratrol at a dose of 50 mg/kg reduced cerebral ischemia reperfusion damage, brain edema, and BBB malfunction [183]. Outcomes of resveratrol on IS from in vivo studies are summarized in Table 1.

**Table 1:** Animal experiments with resveratrol in the brain injury.

Reference number	Animal model	Type of brain injury	Root of administration	Time of administration	Dose	Evaluated parameters	Main findings
[184]	Male and female C57BL/6 mice (age 10–11 weeks)	Transient MCAO	IV	3 or 6 h after MCAO	1, 2.5, or 5 mg/kg resveratrol	Infarct size	▶Male: Res _5_ _mg/kg_ and Res _2.5_ _mg/kg_ decreased infarct size ▶Female: Res _1_ _mg/kg,_ Res _2.5_ _mg/kg,_ and Res _5_ _mg/kg_ decreased infarct size. ▶Res _5_ _mg/kg / 3 or 6 h_ decreased infarct size in all mice
						RT-PCR (Expression levels of IL-1β and TNF-α)	▶ Resveratrol decreased IL-1β mRNA and TNF-α mRNA levels
						. Western blotting (IL-1β, TNF-α, and Iba1)	▶ Resveratrol decreased IL-1β protein, TNF-α protein and Iba1protein levels
						ROS production in the cortex and striatum	▶ Resveratrol decreased ROS production in the cortex and striatum
[185]	Male C57BL/6 mice, aged 10–11 weeks	MCAO	IV injection by tail vein	3 h after MCAO	5 mg/kg	Infarct size	▶ Resveratrol decreased infarct size in total ▶ Resveratrol decreased infarct size in the cortex ▶ Resveratrol did not decrease infarct size in the striatum
						RT-PCR (Expression levels of PGC1a, pCREB UCP2, SOD2, and Bcl-2)	▶ Resveratrol increased PGC1a ▶ resveratrol increased pCREB levels ▶ Resveratrol increased UCP2, SOD2 and Bcl-2 levels
						Western blotting (SIRT1, pAkt, pERK1/2, pp38 and PGC1a protein levels)	▶ Resveratrol increased SIRT1 levels ▶ Resveratrol increased PAkt levels ▶ Resveratrol increased pERK1/2 levels compared to the control group but not the vehicle group ▶ Resveratrol increased p38 levels ▶ Resveratrol increased PGC1a levels
[186]	Male Wistar rats	MCAO	IP	For 10 days, once a day before MCAO	125, 250, 500 μg/kg/day nanostructured lipid carriers (NLC) containing resveratrol	Behavioral tests: - Flexion test - Spontaneous motor activity - Grip strength - Rota-Rod test - Morris water maze	▶ Resveratrol improved flexion test in a dose dependent manner (higher doses were associated with better outcomes) ▶ Resveratrol improved Spontaneous motor activity in a dose dependent manner (higher doses were associated with better outcomes) ▶ Resveratrol improved Rota-Rod test in a dose dependent manner (higher doses were associated with better outcomes) ▶ In the Morris water maze, resveratrol decreased Escape latency time and increased the time spent in the target area
						Infarction volume	▶ Resveratrol decreased infarct volume in a dose dependent manner
						Biochemical estimations	▶ Resveratrol decreased LPO content in a dose dependent manner ▶ Resveratrol increased GSH content in a dose dependent manner ▶ Antioxidant enzymes (GPx, GR, GST, SOD and Catalase) were increased by resveratrol in a dose dependent manner ▶ Activity of Na + -K + ATPase were increased by resveratrol in a dose dependent manner ▶ Apoptotic markers (Caspase- 3 and 9) were decreased by Res_500_ ▶ Cytokines estimation (Level of IL-1β, IL-6, and TNFɑ) were decreased by Res_500_
[78]	Male wild-type C57BL/6 (B6) mice	MCAO	Oral gavage/IP	For 3 consecutive days before MCAO	30 mg/kg/day DMF	mNSS score Rotarod test Grip strength	▶ Resveratrol decreased mNSS scores ▶ Resveratrol increased Latency time ▶ Resveratrol increased grip strength
						Infarct size	▶ Resveratrol decreased infarct size
[170]	Male rats	Single MCAO (30 min) Recurrent MCA (2 × 30 min MCAO)	Oral gavage	For 3 days prior to the MCAO For 3 days, the first dose was administered 1 h following the initial stroke and the final dose 1 h prior to the second stroke	25 mg/kg resveratrol	Measurement of BBB Permeability	▶ Resveratrol decreased BBB permeability
						Measurement of brain water content	▶ Resveratrol decreased brain water content
						Histological Assessment of Cell Death with H&E staining	▶ Resveratrol decreased cell death
						glial activation	▶ Resveratrol decreased glial activation
						microglial/macrophage activation	▶ Resveratrol decreased microglial/macrophage activation
						nitrosative stress	▶ Resveratrol decreased nitrosative stress
[187]	Sprague-Dawley rats	MCAO	IP	For 7 consecutive days after MCAO	10 mg/kg and 100 mg/kg of resveratrol	Neurological deficits score	▶ Res _100_ decreased neurological deficit scores
						Infarct Volume	▶ Res _100_ decreased infarct volume
						Measurement of brain water content	▶ Res _100_ decreased brain water content
						Measurement of BBB Permeability	▶ Res _100_ decreased BBB permeability
						Myeloperoxidase (MPO) enzymatic activity	▶ Res _100_ decreased MPO enzymatic activity
						Western blot (TLR4 and NF-κB p65 protein levels)	▶ Resveratrol decreased TLR4 levels in a dose dependent manner ▶ Res _100_ decreased NF-κB p65 levels
						RT-PCR (expression levels of Cyclooxygenase-2 (COX-2) and MMP-9 in ischemic brain tissues and also blood levels of proinflammatory cytokines (TNF-α and IL-1β)	▶ COX-2 and MMP-9 were decreased by resveratrol in a dose dependent manner ▶ TNF-α and IL-1β were decreased by resveratrol in a dose dependent manner
[188]	Male mice	MCAO	IP	5 min before reperfusion	30 mg/kg/day	Morris Water Maze	▶ Resveratrol decreased Escape latency time ▶ Resveratrol increased time spent in target area
						Rota Rod test	▶ Resveratrol increased latency time
						Inclined beam walking test (Motor performance deficit)	▶ Resveratrol decreased motor performance deficit
						Infarct volume	▶ Resveratrol decreased infarct volume
						Brain biochemical parameters (TBARS, GSH, SOD, AChE)	▶ Resveratrol decreased TBARS levels ▶ Resveratrol increased GSH levels ▶ Resveratrol increased SOD levels ▶ Resveratrol decreased AChE levels
[189]	Male Sprague-Dawley rats	MCAO	Abdominal injection	For 21 days after MCAO	5 mg/kg/day	Neurological Function Balance beam scores Rota Rod test	▶ Resveratrol improved neurological function ▶ Resveratrol decreased balance beam scores ▶ Resveratrol decreased latency time in Rota Rod test
						RT-PCR (mRNA expression levels of BDNF and TrkB(	▶ Resveratrol increased mRNA expression levels of BDNF and TrkB
						Western Blotting (BDNF and TrkB protein levels)	▶ Resveratrol increased BDNF and TrkB protein levels
[190]	Male Charles foster rats	MCAO	IP	30 min prior to MCAO (Pre) or 2 h post MCAO (Post)	40 mg/kg	Neurological deficits score	▶ Res _Pre_ decreased neurological deficit scores compared to all groups ▶ Res _Post_ decreased neurological deficit scores compared to the MCAO group
						Infarct Volume	▶ Res _Pre_ decreased infarct volume compared to all groups ▶ Res _Post_ decreased infarct volume compared to the MCAO group
						Brain Edema	▶ Res _Pre_ decreased brain edema compared to all groups ▶ Res _Post_ decreased brain edema compared to the MCAO group
						Biochemical assay (nitrite and malondialdehyde (MDA) levels)	▶ Res _Pre_ decreased nitrite and MDA levels compared to all groups but sham ▶ Res _Post_ decreased brain edema compared to the MCAO group
						Activity of MMP-2 and MMP-9 in cortical and striatal regions	▶ Res _Pre_ decreased MMP-2 and MMP-9 activity in cortical and striatal regions compared to all groups but sham ▶ Res _Post_ decreased MMP-2 and MMP-9 activity in cortical and striatal regions compared to the MCAO group
[191]	C57Bl/6J WT or Nrf2−/− male mice	MCAO	IP	48 h before MCAO	10 mg/kg/day	Infarct Volume	▶ Resveratrol decreased infarct volume in the WT MCAO group ▶ Resveratrol did not decrease infarct volume in Nrf2 −/− mice compared to the control group
						Respiratory control index	▶ Resveratrol decreased Respiratory control index in the WT MCAO group but did not have an effect in the Nrf2 −/− group
[179]	Sprague-Dawley male rats	MCAO	Oral gavage	At 2, 24,48 and 72 h after MCAO	30 mg/kg/day	Neurological deficits score	▶ Resveratrol decreased Neurological deficits scores
						BWC	▶ Resveratrol decreased BWC
						Corner test	▶ Resveratrol decreased Number of right turns
						Infarct Volume	▶ Resveratrol decreased Infarct Volume
						Western Blotting (phospho-Akt and phospho-GSK-3β protein levels in cerebral cortex)	▶ Resveratrol increased *p*-GSK-3β levels ▶ Resveratrol increased *p*-Akt levels
[192]	Male Sprague-Dawley rats	MCAO	IP	For 7 consecutive days after MCAO and once prior to MCAO	30 mg/kg once daily	Neurological deficits score	▶ Resveratrol decreased Neurological deficits scores
						Infarct Volume	▶ Resveratrol decreased infarct volume
						Western Blotting Analysis (AK2, STAT3, AKT, mTOR, *p*-JAK2, p-STAT3, *p*-AKT, *p*-mTOR, BCL-2, BAX, and cleaved caspase-3 protein levels)	▶ Resveratrol decreased *p*-AKT levels ▶ Resveratrol had no effect on AKT levels ▶ Resveratrol decreased *p*-mTORl levels ▶ Resveratrol had no effect on mTOR levels ▶ Resveratrol increased *p*-JAK2 levels ▶ Resveratrol had no effect on JAK2 levels ▶ Resveratrol increased p-STAT3 levels ▶ Resveratrol had no effect on STAT3 levels ▶ Resveratrol increased BCL-2 levels ▶ Resveratrol decreased Bax levels ▶ Resveratrol decreased cleaved caspase-3 levels
						RT-PCR (mRNA expression levels of BCL-2, BAX, and cleaved caspase-3 (	▶ Resveratrol increased BCL-2 levels ▶ Resveratrol decreased Bax levels ▶ Resveratrol decreased cleaved caspase-3 levels
						Immunocytochemistry	▶ Resveratrol increased BCL-2 levels ▶ Resveratrol decreased BAX-positive cells ▶ Resveratrol decreased cleaved caspase-3 levels
						TUNEL Staining	▶ Resveratrol decreased TUNEL-positive cells
[193]	Male Sprague-Dawley rats	MCAO OGD	IP	For 7 consecutive days before MCAO and 30 min before ischemia	30 mg/kg/day	Neurobehavioral tests: - Longa score - Bederson score - mNSS	▶ Resveratrol decreased Longa scores ▶ Resveratrol decreased Boderson scores ▶ Resveratrol decreased mNSS scores
						Infarct Volume	▶ Resveratrol decreased infarct volume
						TUNEL Staining	▶ Resveratrol decreased TUNEL-positive cells
						Immunocytochemistry and Immunohistochemistry	▶ Resveratrol decreased TUNEL^+^/NeuN + cells ▶ Resveratrol increased Shh+/NeuN + cells ▶ Resveratrol increased NeuN ^+^ cells ▶ Resveratrol increased OD value of primary cortical neurons
						RT-PCR (mRNA expression levels of Shh, Ptc-1, Smo, and Gli-1)	▶ Resveratrol increased mRNA expression levels of Shh, Ptc-1, Smo, and Gli-1
	Male Sprague-Dawley rats	MCAO	IP	Starting at 3 h after reperfusion and lasting for 4 days	30 mg/kg/day	Neurological deficits score	▶ Resveratrol decreased Neurological deficits scores
						Infarct Volume	▶ Resveratrol decreased infarct volume
						Measurement of brain water content	▶ Resveratrol decreased brain water content
						Histological Assessment with H&E staining	▶ Resveratrol increased cortical neurons number
						Western Blotting Analysis (caspase-3, cleaved cas-pase3, Bcl-2, Bax protein levels)	▶ Resveratrol increased Bcl-2 levels ▶ Resveratrol decreased Bax levels ▶ Resveratrol decreased Cleaved caspase-3 levels ▶ Resveratrol decreased total caspase-3 levels
						Myeloperoxidase (MPO) level	▶ Resveratrol decreased MPO levels
						TNF-α level	▶ Resveratrol decreased TNF-α levels
[194]	Female C57BL/6 mice (aged 20 months)	MCAO	Oral gavage	For 10 days starting 7 days before MCAO	0.1 mg/kg	Serum cytokine (interleukin (IL)-1β and tumor necrosis factor-α (TNF-α)) levels	▶ Resveratrol decreased IL-1β levels ▶ Resveratrol decreased TNF-α levels
						Neurobehavioral tests: Neurological deficits score	▶ Resveratrol and vehicle group did not differ significantly in neurological deficit scores

						Wire suspension test	▶ Resveratrol increased wire suspension test scores
						Grip strength test	▶ Resveratrol and vehicle group did not differ significantly in the grip strength test
						Infarct Volume	▶ Resveratrol decreased total infarct volume ▶ Resveratrol decreased infarct volume in the cortex ▶ Resveratrol and vehicle group did not differ significantly in the striatum infarct volume
						RT-PCR (mRNA expression levels of IL-1β, IL-6, TNF-α)	▶ Resveratrol decreased IL-1β levels ▶ Resveratrol decreased TNF-α levels
						Western Blotting Analysis (IL-1β, claudin-5, occludin, TNF-α protein levels)	▶ Resveratrol decreased IL-1β levels ▶ Resveratrol decreased TNF-α levels ▶ Resveratrol increased claudin-5 and occludin levels
						Adipocyte size measurement	▶ Resveratrol decreased Adipocyte size
[180]	Female Wistar rats	Intracerebroventricular (ICV) collagenase induced intracerebral hemorrhage (ICH) oral gavage		For 21 days	5, 10 and 20 mg/kg	Neurobehavioral tests: -Spontaneous motility	▶ Resveratrol increased spontaneous motility in a dose dependent manner
						-Horizontal bar test	▶ Resveratrol increased horizontal bar test scores in a dose dependent manner
						-Righting reflex	▶ Res_20_ increased righting reflex
						-Forelimb flexion	▶ Resveratrol increased forelimb flexion scores in a dose dependent manner
						-Rotarod	▶ Resveratrol increased rotarod scores in a dose dependent manner
						-Cylinder test	▶ Resveratrol increased cylinder test scores in a dose dependent manner
						-Forced swim test	▶ Res _20_ decreased immobility time
						-Assessment of total locomotor activity	▶ Resveratrol increased total locomotor activity in a dose dependent manner
						-Assessment of mechanical hyperalgesia	▶ Res_20_ and Res_10_ increased mechanical hyperalgesia
						-Assessment of allodynia	▶ Resveratrol increased allodynia in a dose dependent manner
						-Morris water maze test -Maze retention probe trial	▶ Resveratrol decreased escape latency time in a dose dependent manner ▶ Resveratrol increased time spent in target quadrant in a dose dependent manner
						Extent of lipid peroxidation Reduced glutathione Catalase activity SOD activity Nitrite levels	▶ Resveratrol decreased extent of lipid peroxidation in a dose dependent manner ▶ Resveratrol increased reduced glutathione levels in a dose dependent manner ▶ Resveratrol increased catalase activity ▶ Resveratrol increased SOD activity ▶ Res_20_ and Res_10_ decreased nitrite levels
						Quantification of TNF-α	▶ Res_20_ and Res_10_ decreased TNF-α levels
[174]	Elderly male Wistar rats aged 19–21 months	single MCAO and double MCAO	Oral gavage	For 3 days prior to MCAO	25 mg/kg	Neurobehavioral tests: Horizontal ladder task Cylinder task	▶ Resveratrol decreased stepping error rate in single/double MCAO ▶ Resveratrol decreased asymmetry score in single/double MCAO
						Infarct Volume (TTC staining and MRI)	▶ Resveratrol decreased infarct volume in single/double MCAO
						SIRT1 activity and expression	▶ Resveratrol increased SIRT1 activity in Single/double MCAO ▶ Resveratrol and vehicle group did not differ in SIRT1 expression in Single/double MCAO
						Measurement of AMPK activities	▶ Resveratrol increased AMPK activity
						NAD+/NADH ratio	▶ Resveratrol increased NAD+/NADH ratio
						ATP levels	▶ Resveratrol increased ATP levels
[156]	Male Wistar rats (3–4 months old)	MCAO	IP	For 7 consecutive days before MCAO and 30 min before ischemia	30 mg/kg	Neurological deficits score	▶ Resveratrol decreased neurological deficit scores
						Infarct Volume	▶ Resveratrol decreased infarct volume
						SOD and MDA levels in the Hippocampus	▶ Resveratrol increased SOD levels ▶ Resveratrol decreased MDA levels
						TUNEL Staining	▶ Resveratrol decreased number of TUNEL-positive cells
						Western Blotting Analysis (LC3, Bcl-2 and Bax protein levels)	▶ Resveratrol increased Bcl-2 and Bax levels ▶ Resveratrol increased LC3-II and LC3-I levels
						LDH and CK Release Assay	▶ Resveratrol decreased LDH and CK levels
[157]	Female Sprague-Dawley rats	Global cerebral infarction (GCI, bilateral common carotid artery occlusion)	IP	Single dose after Global cerebral infarction	20 and 40 mg/kg	Serum Bcl-2 levels Serum Annexin V levels Serum p53 levels	▶ Resveratrol increased Bcl-2 levels in a dose dependent manner ▶ Res_40_ and Res_20_ decreased serum Annexin V levels ▶ Resveratrol decreased serum p53 levels in a dose dependent manner
[195]	Male Sprague-Dawley rats	MCAO	Not mention	For 7 days before MCAO and 30 min before ischemia	30 mg/kg/day	Neurologic deficit scores: Longa Score, modified Bederson Score	▶ Resveratrol decreased longa scores ▶ Resveratrol decreased modified bederson scores
						Immunohistochemistry	▶ Resveratrol decreased number of GFAP + cells
[181]	Postnatal day 7 (P7) rat pups of both genders	Neonatal Hypoxic-Ischemic (HI)	Oral (in the drinking water)	For: A) 2 weeks (last week of gestation + first week of lactation (GL)) B) 1 week (last week of gestation (G)) C) 1 week (first week of lactation (L)) D) 1 week after the HI (C)	0.15 mg/kg/day.	MRI	▶ Res L and Res GL decreased brain lesion volume
						Neurobehavioral tests: -Righting Reflex -Modified Neurological Severity Score (mNSS)	▶ Res G, Res C, Res L, and Res GL decreased righting reflex ▶ Res G, Res C, Res L, and Res GL decreased mNSS scores
						-Novel Object Recognition Test	▶ Res C, Res L, Res GL increased novel object recognition test scores
						RT-PCR (mRNA expression levels)	▶ Res GL increased SIRT1, Bcl2, and SOD2 levels in contralateral hemisphere ▶ Res GL increased SIRT1 and Bcl2 levels in ipsilateral hemisphere ▶ Res GL increased SOD2 in ipsilateral hemisphere
						Western blot analysis (MCT1, MCT2, LDHa, LDHb, GLAST, GLT1, and the Na+/K + -ATPase α2 subunit protein levels)	▶ Res GL increased MCT1, MCT2, LDHa, LDHb, GLAST, GLT1, and Na+/K + -ATPase α2 subunit in contralateral hemisphere ▶ Res GL increased MCT1, MCT2, LDHa, LDHb, GLAST, GLT1, and Na+/K + -ATPase α2 subunit in ipsilateral hemisphere
						Nissl Staining	▶ Res GL and HI group did not differ significantly in Cell viability in contralateral hemisphere ▶ Res GL increased Cell viability in ipsilateral hemisphere
[182]	Seven-day-old Sprague–Dawley rat pups	Neonatal Hypoxic-Ischemic (HI)	IP	For seven consecutive days before HI	20 or 40 mg/kg	Infarct Volume	▶ Res_40_ and Res_20_ decreased infarct volume
						BWC	▶ Res_40_ and Res_20_ decreased BWC
						Lipid peroxidation and antioxidant status	▶ Resveratrol increased antioxidant enzymes (GPx, SOD and Catalase) in a dose dependent manner ▶ Resveratrol decreased MDA levels in a dose dependent manner
						Assessment of inflammatory markers	▶ Resveratrol decreased TNF-α, IL-β, IL-6, and NF-κB p65 levels in a dose dependent manner
						Immunoblot analysis	▶ Resveratrol increased Nrf2 and HO-1 levels in a dose dependent manner
						Immunohistochemistry	▶ Resveratrol increased number of Nrf2 positive cells in a dose dependent manner ▶ Resveratrol increased HO-1 positive cells in a dose dependent manner
[171]	Mice	MCAO	IP	30 min after the beginning of the reperfusion	680 and 6800 μg/kg	Infarct Volume	▶ Res_6800_ decreased infarct volume
						General neurological scale	▶ Resveratrol and vehicle group did not differ in general neurological scale
						Focal neurological scale	▶ Resveratrol and vehicle group did not differ in focal neurological scale
[172]	Adult male Wistar rats	MCAO	IP	for 21 days	20 mg/kg/day trans resveratrol	Neurobehavioral tests: -Grip test	▶ Resveratrol increased grip test scores
						-Rota rod	▶ Resveratrol increased latency time
						-Closed field activity test (Spontaneous locomotor activity)	▶ Resveratrol increased spontaneous locomotor activity
						Estimation of oxidative stress markers	▶ Resveratrol decreased MDA levels ▶ Resveratrol decreased Glutathione levels
						Infarct Volume	▶ Resveratrol decreased infarct volume
[196]	Adult male Balb/C mice	MCAO	Oral gavage	For 7 days before MCAO	50 mg/kg	Infarct Volume	▶ Resveratrol decreased infarct volume
						Western blot analysis (MMP-9 protein levels)	▶ Resveratrol decreased MMP-9 protein levels
						SDS–. PAGE zymogram	▶ Resveratrol and vehicle group did not differ in MMP-2 levels ▶ Resveratrol decreased MMP-9 levels
						RT–PCR analysis (MMP-9 mRNA)	▶ Resveratrol decreased MMP-9 levels
[153]	Male Wistar rats	MCAO	IV	Twice: 15 min pre-occlusion and at the time of reperfusion (2 h post-occlusion)	10^−7^ g/kg	ATP level in hippocampus	▶ Resveratrol increased ATP levels in the hippocampus
						Mitochondrial respiratory chain complexes (I–IV)	▶ Resveratrol increased Mitochondrial respiratory chain complexes (I–IV)
						Western blot analysis for cytochrome *c*	▶ Resveratrol decreased cytochrome *c* levels
						Quantification of heat stress protein (Hsp70) by ELISA	▶ Resveratrol increased Hsp70 levels
						Quantification of metallothionein (MT) by ELISA	▶ Resveratrol increased MT levels
						BWC	▶ Resveratrol decreased BWC
						Infarct Volume	▶ Resveratrol decreased infarct volume
						Neurological severity score Flexion test Spontaneous movement activity Open field activity	▶ Resveratrol decreased neurological severity scores ▶ Resveratrol decreased flexion test scores ▶ Resveratrol decreased Spontaneous movement activity ▶ Resveratrol increased locomotion ▶ Resveratrol increased average speed ▶ Resveratrol increased distance traveled ▶ Resveratrol decreased resting time
[173]	Sprague-Dawley rats	MCAO	IP	For 7 days before MCAO	200 mg/kg	Infarct Volume	▶ Resveratrol decreased infarct volume
						Neurological deficits score	▶ Resveratrol decreased neurological deficits scores
						Western Blot Analysis	▶ Resveratrol increased protein levels of *p*-CREB ▶ Resveratrol decreased calpain-specific aII-spectrin breakdown products of 145 kDa (SBDP145)
						Quantum Dot-Based Immunofluorescence	▶ Resveratrol increased TRPC6 levels
[197]	Adult male Mongolian gerbils	Global forebrain ischemia	IP	A) during a 5-min CCA occlusion and again at 24 h after ischemia B) 5 min after CCA occlusion and again at 24 h after CCA occlusion	30 mg/kg	Histological Assessment with cresyl violet staining	▶ Resveratrol increased the number of live neurons in hippocampal CA1 ▶ Resveratrol decreased GFAP-positive astrocytes ▶ Resveratrol decreased Isolectin-B4 positive cells
[198]	Male Sprague-Dawley rats	MCAO	IP	For 7 days prior to MCAO	50 mg/kg	Neurological deficits score	▶ M Resveratrol increased neurological deficit scores
						Infarct Volume	▶ Resveratrol decreased infarct volume
						Flow Cytometry for Tregs	▶ Resveratrol increased frequency of Tregs ▶ Resveratrol increased number of Tregs
						Levels of IL-6, IL-10 and TNF-α	▶ Resveratrol decreased Plasma IL-6 and TNF-α Levels: MCAO + vehicle > MCAO + Res ▶ Resveratrol increased Plasma IL-10 levels ▶ Resveratrol decreased ischemic hemisphere IL-6and TNF-α Levels ▶ Resveratrol increased ischemic hemisphere IL-10 Levels
						Functional Assay of Treg	▶ Resveratrol decreased proliferation index of PHA-activated spleen lymphocytes ▶ Resveratrol increased Level of IL-10 in the supernatant of purified Tregs
[199]	Adult diabetic Wistar rats	Global cerebral infarction (bilateral common carotid artery occlusion)	IP	5 min before reperfusion	5, 10, 20, and 30 mg/kg	Infarct Volum	▶ Resveratrol decreased infarct volume in a dose dependent manner
						Estimation of oxidative stress and inflammation markers	▶ Resveratrol decreased MDA levels ▶ Resveratrol increased SOD increased ▶ Resveratrol increased CAT levels ▶ Resveratrol decreased MPO levels ▶ Resveratrol decreased TNF-α levels ▶ Resveratrol decreased IL-6 levels ▶ Resveratrol increased IL-10 levels
[222]	Adult male mice (C57BL/6)	MCAO	Oral gavage	One time 2 h before MCAO (acute regimen) or once daily for 7 days (chronic regimen)	5, 10, and 20 mg/kg	Infarct Volume	▶ Resveratrol decreased infarct volume
[176]	Adult male Sprague Dawley rats	MCAO	IP	Once a day for 7 days before MCAO	50 mg/kg	Neurological deficits score	▶ Resveratrol decreased neurological deficit scores
						Infarct Volume	▶ Resveratrol decreased infarct volume
						Western Blot Analysis (Bcl-2, Bax, caspase-3, and IL-1β, LC3I, LC3II, and Beclin-1 protein expression)	▶ Resveratrol increased Bcl-2/Bax levels ▶ Resveratrol decreased caspase-3 and IL-1β levels ▶ Resveratrol increased LC3 II/LC3 I levels ▶ Resveratrol increased Beclin-1 levels
[200]	Adult male Sprague Dawley rats	MCAO	IP	For 7 days after MCAO	30 mg/kg	Neurological deficits score	▶ Resveratrol decreased neurological deficit scores
						Infarct Volume	▶ Resveratrol decreased infarct volume
						Monitoring of neurotransmitter and neuromodulator	▶ Resveratrol decreased Glu and Asp ▶ Resveratrol increased GABA, Gly and Tau ▶ Resveratrol decreased neuromodulator PEA, D-Ser and excitotoxic index
[177]	Adult male Sprague Dawley rats	MCAO	IP	Once a day for 6 days before MCAO	10,20 and 30 mg/kg	Neurological deficits score	▶ Resveratrol decreased neurological deficit scores in a dose dependent manner
						Infarct Volume	▶ Resveratrol decreased infarct volume in a dose dependent manner
						Western Blot Analysis (SIRT1 and *p*-AMPK protein expression)	▶ Resveratrol increased SIRT1 and *p*-AMPK
						RT-PCR (PDEs and SIRT1 mRNA expression levels)	▶ Resveratrol increased SIRT1 ▶ Resveratrol decreased PDE4A ▶ Resveratrol and vehicle group showed no significant difference in PDE7A levels
						Brain levels of ATP	▶ Resveratrol increased brain levels of ATP
						Quantitative assay for cAMP	▶ Resveratrol increased cAMP levels
[178]	Adult male Sprague Dawley rats	MCAO	Internal carotid artery (ICA)		1,5 and 10 mg/kg resveratrol encapsulated into polymeric nanoparticles (RES-NPs)	Neurological behavior assessment	▶ Res_10_ and Res_5_ decreased neurological behavior assessment
						Infarct Volume	▶ Resveratrol decreased infarct volume
						Measurement of BBB Permeability	▶ Resveratrol decreased BBB permeability
						Measurement of brain water content	▶ Resveratrol decreased brain water content
						Western Blot Analysis (BDNF, Bax, cleaved caspase-3 and Bcl-2 protein expression)	▶ Resveratrol increased Bcl-2/Bax levels ▶ Resveratrol decreased Caspase-3 levels ▶ Resveratrol increased BDNF levels
						MDA levels of the cerebral cortex	▶ Resveratrol decreased MDA levels
						TUNEL staining	▶ Resveratrol decreased number of TUNEL positive cells
[201]	Adult male Sprague Dawley rats	MCAO	IP	At 1, 4, 6, 12, or 24 h before MCAO	30 mg/kg	Infarct Volume	▶ Res 1h and Res 4h decreased infarct volume
						Morris water maze test	▶ Res 1h and Res 4h decreased latency
						Western Blot Analysis	▶ Resveratrol increased pERK/ERK ▶ Resveratrol increased pCREB/CREB
[202]	Adult male Sprague Dawley rats	Transient and permanent MCAO	IV	30 min prior to MCAO	2 × 10^−7^, 2 × 10^−6^, 2 × 10^−5^, 2 × 10^−4^, 2 × 10^−3^ mg/kg	Infarct Volume	▶ Res 2 × 10^−3^ and Res 2 × 10^−4^ decreased infarct volume
[203]	Adult male Sprague Dawley rats	MCAO	IP	Once a day for 7 days and 30 min before MCAO	30 mg/kg	Neurobehavioral tests (at 1 day, 7 days and 14 days after MCAO): -Longa score -Bederson score -mNSS	▶ Resveratrol decreased longa scores ▶ Resveratrol decreased bederson scores ▶ Resveratrol decreased mNSS scores
						RT-PCR (Shh and Gli-1 mRNA expression levels)	▶ Resveratrol increased Shh and Gli-1
						Immunohistochemistry (number of Tba-1 and GFAP + cells)	▶ Resveratrol decreased number of Tba-1 and GFAP + cells
[204]	Adult male Sprague Dawley rats	MCAO	IP	For 7 days before surgery and 30 min before MCAO	15 and 30 mg/kg	Neurological deficits score	▶ Resveratrol decreased neurological deficit scores in a dose dependent manner
						Infarct Volume	▶ Resveratrol decreased infarct volume in a dose dependent manner
						Measurement of brain water content	▶ Res _30_ and Res _15_ decreased brain water content
						MDA levels and SOD activity	▶ Res _30_ and Res _15_ decreased MDA levels ▶ Res _30_ and Res _15_ increased SOD activity
						Western Blot Analysis (Nrf2 and heme oxygenase-1 (HO-1) expression levels)	▶ Res _30_ and Res _15_ increased Nrf2 and HO-1 levels
						RT-PCR (Nrf2 and heme oxygenase-1 (HO-1) mRNA expression levels)	▶ Res _30_ and Res _15_ increased Nrf2 and HO-1 levels
						TUNEL staining	▶ Res _30_ and Res _15_ decreased number of TUNEL positive cells
						Immunohistochemistry (protein expression of Caspase-3)	▶ Res _30_ and Res _15_ decreased Caspase-3 levels
						-Rota-rod test	▶ Resveratrol increased Rota rod test scores
						-Water maze	▶ Resveratrol decreased reference memory (Latency time) ▶ Resveratrol decreased working memory (Latency time)
						Histological damage score with H&E	▶ Resveratrol decreased Histological damage scores
						Thickness of corpus callosum at the level of dorsal hippocampus in three sites (ipsilateral, center, contralateral) with Luxol fast blue stain staining	▶ Resveratrol increased thickness of corpus callosum at the level of dorsal hippocampus in ipsilateral, center and contralateral
[205]	Adult male C57BL/6 mice	MCAO	IP	For 3 days after MCAO	200 mg/kg	Behavioral Test	▶ Resveratrol increased neurologic scores
						Infarct volume measurement	▶ Resveratrol decreased infarct volume
						Histological score with H&E)Gut Injury Score(	▶ Resveratrol decreased Gut injury scores
						ELISA for Albumin Quantification	▶ Resveratrol decreased albumin quantification
						Measurement of BBB Permeability	▶ Resveratrol decreased BBB permeability
						Western Blotting (zonula occludens 1 (ZO-1, occludin, and claudin-)	▶ In the small intestine, resveratrol increased ZO-1, occludin, and claudin-1
						RT-PCR (pro-inflammatory cytokines and anti-inflammatory cytokine mRNA expression levels)	▶ In the peri-infarct region, resveratrol decreased IL-17A, TNF-α, and IFN-γ but increased IL-4 and IL-10 levels ▶ In the peri-infarct region and serum levels, resveratrol decreased IL-17A, TNF-α, and IFN-γ but increased IL-4 and IL-10 levels
[183]	Adult Male Sprague-Dawley rat	MCAO	IP	At the onset of reperfusion	50 mg/kg	Neurological Deficit Sore	▶ Resveratrol increased neurological deficit scores
						Infarct Volume	▶ Resveratrol decreased infarct volume
						Measurement of BBB Permeability	▶ Resveratrol decreased BBB permeability
						Measurement of Brain Water Content	▶ Resveratrol decreased brain water content
						TUNEL Staining	▶ Resveratrol decreased number of TUNEL positive cells
						Western Blot (MMP-9 and TIMP-1 expression levels)	▶ Resveratrol decreased MMP-9 levels ▶ Resveratrol increased TIMP-1 levels
						Gelatin Zymography (enzymatic activity of MMP-9 and TIMP-1)	▶ Resveratrol decreased MMP-9 levels ▶ Resveratrol increased TIMP-1 levels
[206]	Adult Male Sprague-Dawley rat	MCAO		Before MCAO	20 mg/kg	Infarct Volume	▶ Resveratrol decreased infarct volume
						Measurement of BBB Permeability	▶ Resveratrol decreased BBB permeability
						Measurement of Brain Water Content	▶ Resveratrol decreased brain water content
						TUNEL Staining	▶ Resveratrol decreased number of TUNEL positive cells
						RT-PCR (YAP and TAZ mRNA expression levels(	▶ Resveratrol increased YAP and TAZ expression levels
[207]	Male Wistar rats	MCAO	IV	At the onset of reperfusion	1.9 mg/kg	Neurological performance	▶ Resveratrol increased neurological performance
						Infarct Volume	▶ Resveratrol decreased infarct volume
						Measurement of BBB Permeability	▶ Resveratrol decreased BBB permeability
						Measurement of Brain Water Content	▶ Resveratrol decreased brain water content
						Electrophoretic Mobility Shift Assay (SP1 binding activity)	▶ Resveratrol decreased SP1 binding activity
						RT-PCR (SUR1 and AQP4 mRNA expression levels)	▶ Resveratrol decreased SUR1 and AQP4 levels
						Western blot (SUR1 and AQP4 expression levels)	▶ Resveratrol decreased SUR1 and AQP4 levels
[208]	Adult Male Sprague-Dawley rat	MCAO	IP	At the onset of reperfusion	100 mg/kg	Infarct Volume	▶ Resveratrol decreased infarct volume
						Measurement of Brain Water Content	▶ Resveratrol decreased brain water content
						NDS	▶ Resveratrol decreased NDS
						Western blot (Sirt1, p62, C3B-II/LC3B–I and NLRP3 inflammasom expression levels)	▶ Resveratrol increased Sirt1 levels ▶ Resveratrol decreased p62 levels ▶ Resveratrol increased C3B-II/LC3B–I levels ▶ Resveratrol decreased NLRP3 inflammasom levels
[209]	Male Wistar rats	MCAO	IV	At the onset of reperfusion	10^(−8)^ g/kg, 10^(−7)^ g/kg, and 10^(−6)^ g/kg	Infarct Volume Measurement of Brain Water Content	▶ Resveratrol decreased infarct volume in a dose dependent manner ▶ Res 10^(−7)^ and Res 10^(−6)^ decreased brain water content
[210]	Adult male Sprague-Dawley rats	MCAO	IP	At 10 min prior to MCAO in addition to at 0 and 20 h following reperfusion.	20 mg/kg	Neurological deficits	▶ Resveratrol decreased neurological deficit
						Infarct Volume	▶ Resveratrol decreased infarct volume
						Measurement of Brain Water Content	▶ Resveratrol decreased brain water content
						Lipid peroxidation MDA and SOD activity assay	▶ Resveratrol decreased MDA levels ▶ Resveratrol increased SOD activity
						iNOS activity assay	▶ Resveratrol decreased iNOS levels
						Western blot analysis (AQP4 protein expression)	▶ Resveratrol decreased AQP4 levels
[211]	Adult male Wistar rats	Transient global cerebral ischemia	IP	For 7 days prior to I/R	30 mg/kg	Western blot analysis	▶ Resveratrol decreased GFAP and Isolectina B4 (IB4) levels ▶ Resveratrol increased Cytoplasmatic (CF) fraction of NF-κB (p65) ▶ Resveratrol decreased Nuclear (NF) fraction of NF-κB (p65) ▶ Resveratrol decreased iNOS and COX-2 levels▶ *p*-JNK/JNK levels: IR > IR + Res = sham
[212]	Adult Sprague-Dawley female rats	Transient global cerebral ischemia)bilateral common carotid artery occlusion method(	IP	Before reperfusion	20 and 40 mg/kg	Serum Bcl 2 levels Serum p53 levels Serum Annexin V levels	▶ Resveratrol increased Bcl2 levels in a dose dependent manner ▶ Resveratrol decreased p53 levels in a dose dependent manner ▶ Resveratrol decreased Annexin V levels
[213]	Adult male Sprague–Dawley rats	MCAO	IP	Starting at 3 h after reperfusion and lasting for 4 days	30 mg/kg	Infarct Volume	▶ Resveratrol decreased infarct volume
						Histological examination with H&E	▶ Resveratrol increased number of surviving neurons
						TUNEL staining	▶ Resveratrol decreased numbers of TUNEL-positive cells
						Western blot analysis (Bcl-2 and Bax protein expression)	▶ Resveratrol increased Bcl-2 levels ▶ Resveratrol decreased Bax levels

Res, Resveratrol.

MCAO, Middle cerebral artery occlusion.

HI, Cerebral hypoxia-ischemia model.

ICH, Intracerebral hemorrhage.

IV, Intravenous injection.

IP, Intraperitoneal injection.

BWC, Brain water content.

RT-PCR, Reverse transcription-polymerase chain reaction.

ILs, Interleukins.

TNF-α, Tumor necrosis factor-alpha.

Iba1, Ionized calcium binding adaptor molecule 1.

ROS, Reactive oxygen species.

PGC1a, Peroxisome proliferator-activated receptor-γ coactivator-1α.

pCREB, cAMP-triggered phosphorylation of cAMP response element binding protein.

UCP2, Uncoupling protein 2.

Bcl-2, B-cell lymphoma-2.

BAX, Bcl-2-associated X protein.

MPO, Myeloperoxidase.

SIRT1, Sirtuin 1.

pERK1/2, phospho-extracellular signal regulated kinase.

PGC1a, Peroxisome proliferator-activated receptor gamma coactivator 1-alpha.

NF-κB, Nuclear factor κB.

TLR4, Toll Like Receptor 4.

COX-2, Cyclooxygenase-2.

MMP-9, Matrix metallopeptidase 9.

TBARS, Thiobarbituric acid reactive substances.

AChE, Acetylcholinesterase.

BDNF, Brain Derived Neurotrophic Factor.

TrkB, Tropomyosin receptor kinase B.

SOD, Superoxide dismutase.

MDA, Malondialdehyde.

GSH, Glutathione.

Nrf2, Nuclear factor erythroid 2–related factor 2.

HO-1, Heme oxygenase-1.

AQP4, Aquaporin-4.

LDH, Lactate dehydrogenase.

Tregs, regulatory T cells.

## 7 *In vitro* studies with resveratrol and stroke

Oxygen glucose deprivation (OGD) is a frequently used technique to mimic stroke and investigate molecular mechanisms underlying the condition. The OGD model is widely used to evaluate the possible therapeutic options for stroke. Pretreatment with resveratrol was able to significantly lower astrocytic activation after OGD/reperfusion *in vitro* [195]. Resveratrol showed beneficial impacts in treating neuronal damage caused by OGD. It up-regulated PPAR-α expression in cultured neurons under OGD conditions, as well as suppressed of MMP-9 mRNA expression [214]. Resveratrol exerts anti-apoptotic actions in OGD, and it contributes to ERK by inhibiting MMP-9 production [215]. ERK plays a key role in almost every cell function and regulates antiproliferative events, including apoptosis [216]. *Faggi* et al. demonstrated that valproate at 1 nmol/mL generated synergistic neuroprotection with resveratrol (3 nmol/mL) in primary neurons exposed to OGD [171]. *Narayanan* et al. concluded that loss of Nrf2 decreased resveratrol potential for neuroprotection and that this pathway plays a crucial role in resveratrol neuroprotective effects [217]. Another *in vitro* model showed that resveratrol inhibits neuronal apoptosis after OGD/reperfusion and boosts Nrf2 activation in a dose dependent manner. Resveratrol administration at different times (pre and post stroke) led to different outcomes (Table 2) [133]. Another study revealed that resveratrol treatment decreased TUNEL positive cells while increasing cell viability through the activation of the sonic hedgehog (Shh) pathway [218]. Another *in vitro* model found that cell viability, SIRT1 activity, AMPK activity, NAD^+^/NADH ratio, and ATP levels were improved with resveratrol [219]. Resveratrol treatment 24 h prior to OGD/reperfusion was able to increase cell viability, decrease cell proliferation and reduce inflammatory cytokines. Expression levels of the Shh proteins (Smo, Ptc-1, and Gli-1) were significantly increased. Resveratrol was also able to inhibit the expression of GFAP, S100β, and Vimentin proteins. These proteins are markers of stress and CNS insult [220,221]. Table 2 summarizes the effects of resveratrol *in vitro* experiments.

**Table 2:** In vitro experiments with resveratrol in ischemic conditions.

Reference number	Type of cell culture	Type of ischemia in-vitro model	Time of exposure to resveratrol	Resveratrol Dose	Evaluated parameters	Main findings
[222]	Cortical neuronal cells isolated from 17-day-old embryos of timed pregnant C57BL/6 mice	OGD/R	For 6 h before OGD/R	25 Μm	MTT assay Western Blot Analysis (HO1 and HO2 protein expression)	▶ Resveratrol increased cell survival ▶ Resveratrol increased HO1levels ▶ Resveratrol and control group did not differ significantly in HO2 levels
[78]	Primary microglia cells	OGD/R			Activation of anti-inflammatory microglia CD206+/Iba-1+ RT-PCR (Expression levels of pro-inflammatory microglia markers (CD11b, CD16) and cytokines (TNF-α, IL-1β, and IL-6) Activation of the CD147/MMP-9 pathway	▶ Resveratrol decreased activation of anti-inflammatory microglia CD206+/Iba-1+ ▶ Resveratrol decreased CD11b, CD16, TNF-α, IL-1β, and IL-6 levels ▶ Resveratrol decreased CD147 levels ▶ Resveratrol decreased MMP-9 levels
[170]	Rat brain endothelial cells	OGD/R	For three consecutive days	100 nM-10 μM	Cell viability	▶ Low doses of Resveratrol increased cell viability whereas higher doses (>1 μM) were associated with reduced cell viability
[191]	Astrocyte cultures	In Vitro Preconditioning	For 2 h, 48 h before harvesting cell lysates	25 μmol/L Resveratrol preconditioning (RPC)	ELISA (Nrf2 DNA binding)	▶ Resveratrol increased Nrf2 DNA binding
					Western Blotting (NAD(P)H-quinone oxidoreductase 1 (NQO-1) protein levels)	▶ Resveratrol increased NQO-1 levels
[133]	Primary culture of rat cortical neurons	OGD/R	Cells were treated with resveratrol: A) Pretreatment (Pre, 24 h prior to OGD, followed by 150 min of OGD and 24 h of reoxygenation) B) Post-treatment (Post, during 24 h of reoxygenation) C) whole-processing (WP) (24 h before and during 150 min of OGD and 24 h of reoxygenation)	10 μmol/L, 20 μmol/L, 40 μmol/L, 60 μmol/L, and 80 μmol/L	LDH Activity	▶ Res_60_ and Res_40_ decreased LDH activity the most
					SOD Activity	▶ Res_60_ and Res_40_ increased SOD activity the most
					TUNEL Staining	▶ Resveratrol decreased TUNEL-positive cells: Normal < OGD/R + Res _PRE_ = OGD/R + Res _WP_ < OGD/R + Res _Post_ = OGD/R
					Measurement of Cell Viability	▶ Resveratrol increased cell viability: Normal > OGD/R + Res _WP_ > OGD/R + Res _PRE_ > OGD/R + Res _Post_ = OGD/R
					Western Blotting Analysis (Bcl-2 and Caspase-3 protein levels)	▶ Resveratrol increased Bcl-2 levels: Normal < OGD/R < OGD/R + Res _PRE_ = OGD/R + Res _Post_ < OGD/R + Res _WP_ ▶ Resveratrol decreased Caspase-3 levels: Normal < OGD/R + Res _WP_ = OGD/R + Res _PRE_ < OGD/R + Res _Post_ < OGD/R
					Immunocytochemistry (Quantification of data for Nrf2, NQO-1, and HO-1 proteins)	▶ Resveratrol increased Nrf-2 levels: Normal < OGD/R < OGD/R + Res _Post_ < OGD/R + Res _PRE_ < OGD/R + Res _WP_ ▶ Resveratrol increased NQO-1: Normal < OGD/R < OGD/R + Res _Post_ = OGD/R + Res _PRE_ < OGD/R + Res _WP_ ▶ Resveratrol increased HO-1 levels: Normal = OGD/R = OGD/R + Res _Post_ < OGD/R + Res _PRE_ < OGD/R + Res _WP_
[193]	Culture of Cortical Neurons	OGD/R	for 24 h before OGD/R	40 μmol/l	Western Blotting Analysis (Gli-1protein levels)	▶ Resveratrol increased Gli-1 levels
					TUNEL Staining	▶ Resveratrol decreased number of TUNEL-positive cells
					CCK-8 Assay for Cell Viability	▶ Resveratrol increased cell viability
[174]	Primary cortical neuron	Single OGD- and double OGD	For 3 h prior to OGD	0.5 μM	Cell viability	▶ Resveratrol increased cell viability
					SIRT1 activity and expression	▶ Resveratrol increased SIRT1 activity
					AMPK activity assay	▶ Resveratrol increased AMPK activity
					Measurement of NAD+/NADH ratio	▶ Resveratrol increased NAD+/NADH ratio
					ATP levels	▶ Resveratrol increased ATP levels
[195]	primary cortical astrocytes cultures	OGD/R	For 24 h prior to OGD	1, 5, 20, 40, 80, 100 μmol/L	CCK-8 Assay for Cell viability	▶ Resveratrol increased cell viability: OGD/R + Res 100 < OGD/R + Res 80 = OGD/R = OGD/R + Res 1 < OGD/R + Res 5 < OGD/R + Res 20 < OGD/R + Res 40 < Normal
					EdU labeling of proliferative cells	▶ Resveratrol decreased proliferative cells
					Western blot analysis (GFAP, S100β and Vimentin, IL-10, IFN-β, TNF-α, IL-1β, Shh, Smo, Ptc-1and Gli-1 protein levels)	▶ Resveratrol decreased GFAP, S100β, TNF-α,IL-1β and Vimentin levels ▶ Resveratrol increased IL-10 and IFN-β ▶ Resveratrol increased Shh, Smo, Ptc-1and Gli-1 levels
					ELISA assay for proinflammatory and anti-inflammatory factors	▶ Resveratrol increased IL-10 and IFN-β proteins levels ▶ Resveratrol decreased TNF-α and IL-1β proteins levels
[171]	Primary cultures of mouse cortical neurons derived from 15-day-old embryonic mice	OGD/R	For 2 h after OGD	3 nmol/mL	Measurement of LDH Release	▶ Resveratrol and vehicle group did not differ in percentage of neurotoxicity
[203]	Cortical neuronal cells	OGD/R	for 24 h before OGD/R	5 μmol/L	Neurite outgrowth of neurons	▶ Resveratrol increased neurite outgrowth of neurons
[214]	Neurons	OGD/R	During the 4 h of OGD and the remaining 20 h	2.5 μM, 5 μM and 10 μM	Cytotoxicity (LDH leakage ratio)	▶ Resveratrol decreased cytotoxicity in a dose dependent manner
					Western blot of PPAR α and γ	▶ Resveratrol increased PPAR α: in a dose dependent manner ▶ Res_10_ and Res_5_ increased PPAR γ levels
					Western blot and RT-PCR of MMP-9	▶ Res_10_ and Res_5_ decreased MMP-9 levels
[215]	Neuronal primary cultures of cerebral cortex were prepared from BALB/c mice embryos (13–15 days)	OGD/R	From OGD until the end of the experiment (24h)	10, 25, 50 and 100 μM	Cell viability assay	▶ Resveratrol increased cell viability
					Apoptosis assay	▶ Resveratrol decreased apoptosis in a dose dependent manner
					Western blot analysis (protein levels of MMP-9, cleaved caspase-3, Bcl-2 and Bax)	▶ Resveratrol decreased MMP-9 levels ▶ Resveratrol decreased cleaved caspase-3 and Bax levels in a dose dependent manner ▶ Resveratrol increased Bcl-2 levels in a dose dependent manner ▶ Resveratrol decreased Extracellular signal-regulated kinase (ERK) in a dose dependent manner
					RT-PCR analysis	▶ Res _100_, Res _50_, and Res_25_ decreased MMP-9 mRNA levels
[223]	PC12 cell line	OGD/R	Pre-treatment (for 24 h prior to OGD) post-treatment (during reoxygenation period (24 h)) whole-treatment (for the entire period of experiment i.e. 24 h prior to OGD, during 6 h of OGD and 24 h of reoxygenation)	5, 10, and 25 μM	Flow cytometric analysis (Reactive oxygen species (ROS) generation)	▶ Pre: Resveratrol decreased ROS ▶ Post: Res_25_ decreased ROS ▶ Whole: Resveratrol decreased ROS
					Glutathione content	▶ Pre: Res_25_ increased glutathione content ▶ Post: Res_25_ increased glutathione content ▶ Whole: Res_25_ increased glutathione content
					Lipid peroxidation	▶ Pre: Res_25_ decreased lipid peroxidation ▶ Post: Res_5_ decreased lipid peroxidation ▶ Whole: Res_25_ decreased lipid peroxidation
					RT-PCR (HIF-1α, Bax, Bcl 2 and Caspase-3 mRNA expression levels)	▶ Resveratrol decreased HIF-1α levels: OGD > OGD + Res _pre_ > OGD + Res _post_ > OGD + Res _whole_ > Control ▶ Resveratrol decreased Bax levels: OGD > OGD + Res _pre_ > OGD + Res _post_ > OGD + Res _whole_ > Control ▶ Resveratrol increased Bcl 2 levels: OGD = OGD + Res _pre_ = OGD + Res _post_ < OGD + Res _whole_ < Control ▶ Resveratrol decreased Caspase-3 levels: OGD + Res _pre_ > OGD > OGD + Res _post_ > OGD + Res _whole_ > Control
					Western blot analysis (HIF-1α, Bax, Bcl 2 and Caspase-3 protein expression)	▶ Resveratrol decreased HIF-1α levels: OGD > OGD + Res _pre_ > OGD + Res _post_ > OGD + Res _whole_ > Control ▶ Resveratrol decreased Bax levels: OGD = OGD + Res _pre_ > OGD + Res _post_ > OGD + Res _whole_ > Control ▶ Resveratrol increased Bcl 2 levels: OGD < OGD + Res _post_ < OGD + Res _pre_ = OGD + Res _whole_ < Control ▶ Resveratrol decreased Caspase-3 levels: OGD = OGD + Res _pre_ > OGD + Res _post_ > OGD + Res _whole_ > Control
[224]	Human SH-SY5Y neuroblastoma cells	OGD/R	For 48 h after OGD	10 μM	MTT (Cell viability) assay	▶ Resveratrol increased cell viability
					ELISA	▶ Resveratrol decreased TNF-α levels ▶ Resveratrol decreased IL-1β levels ▶ Resveratrol decreased IL-18 levels
					RT-PCR (mRNA expression levels)	▶ Resveratrol decreased IKKα levels ▶ Resveratrol decreased IKKβ levels ▶ Resveratrol decreased p65 levels ▶ Resveratrol decreased NLRP3 levels ▶ Resveratrol decreased Caspase 1 levels ▶ Resveratrol increased Nrf2 levels ▶ Resveratrol increased SOD1 and SOD2 levels ▶ Resveratrol increased Gpx levels ▶ Resveratrol increased catalase, and HO-1 levels
					Measuring SOD, Gpx, and GSH Activity	▶ Resveratrol increased SOD, Gpx, and GSH Activity
[225]	Well-differentiated PC12 cells	OGD/R	For 1 h prior to the OGD/R	10 μM	MTT (Cell viability) assay	▶ Resveratrol increased cell vibility
					Detection of apoptotic ratio	▶ Resveratrol decreased apoptosis
					Detection of intracellular ROS production	▶ Resveratrol decreased ROS
					Detection of mitochondrial superoxide generation	▶ Resveratrol decreased mitochondrial superoxide generation
					MDA content	▶ Resveratrol decreased MDA levels
					SOD and catalase activities	▶ Resveratrol increased SOD and catalase activities
					Western blot analysis	▶ Resveratrol decreased cytochrome *c* in cytosol ▶ Resveratrol increased cytochrome *c* in mitochondria ▶ Resveratrol increased Bcl-2/Bax ▶ Resveratrol decreased cleaved caspase-9 ▶ Resveratrol decreased cleaved caspase-3
[226]	Primary cortical neuron culture	OGD/R	For 2 h	0.1, 1.0, and 10.0 μmol/L	Apoptosis assay	▶ Resveratrol decreased neuronal apoptosis
					Measurement of intracellular free calcium concentration	▶ Resveratrol decreased intracellular free calcium
					RT-PCR (mRNA expression levels)	▶ Resveratrol decreased caspases-3 and -12
[227]	Cultures of rat cortical neurons	OGD/R	Immediately prior to reoxygenation, For 2 h	1–10 μM	MTT (Cell viability) assay	▶ Resveratrol increased cell viability
					Western blot analysis	▶Resveratrol decreased caspase-3
					Detection of apoptotic ratio	▶ Resveratrol decreased apoptosis
					Mitochondrial function assays	▶ Resveratrol decreased mitochondrial superoxide ▶ Resveratrol increased TMRE: OGD/R < OGD/R + Res < Normal

OGD/R, Oxygen and glucose deprivation/reoxygenation.

Res, Resveratrol.

NQO-1, NAD(P)H-quinone oxidoreductase 1.

ILs, Interleukins.

TNF-α, Tumor necrosis factor-alpha.

MMP-9, Matrix metallopeptidase 9.

Nrf2, Nuclear factor erythroid 2–related factor 2.

NQO-1, NAD(P)H-quinone oxidoreductase 1.

HO-1, Heme oxygenase-1.

LDH, Lactate Dehydrogenase.

SOD, Superoxide dismutase.

Gpx, Glutathione peroxidase.

Bcl-2, B-cell lymphoma-2.

BAX, Bcl-2-associated X protein.

Gli-1, Glioma-associated oncogene-1.

CCK-8*,* Cell Counting Kit-8.

SIRT1, Sirtuin 1.

AMPK, AMP-activated protein kinase.

GFAP, Glial fibrillary acidic protein.

Shh, Sonic Hedgehog Signaling Molecule.

Smo, Smoothened.

Ptc-1, Patched1.

PPAR, Peroxisome proliferator-activated receptor.

HIF*-*1α*,* Hypoxia-inducible factor-1.

TMRE, Tetramethylrhodamine, ethyl ester.

IKK, inhibitor of nuclear factor-κB (IκB) kinase.

## 8 Clinical trials of resveratrol in ischemic stroke

Few clinical trials that measure the effectiveness of resveratrol on stroke patients. A randomized double-blinded trial assessed the effects of resveratrol on IS patients [228]. Three resveratrol capsules (each containing 170 mg) were given to patients 24h after stroke and were continued for 30 days. Systolic and diastolic blood pressures and the National Institute of Health Stroke Scale (NIHSS) were evaluated at the stroke onset and after discharge. Also, the Barthel index and Modified Rankin Scale (MRS) were performed 3 months following resveratrol consumption. They found that resveratrol had no effects on any of the factors mentioned in IS patients compared to the placebo group. Another study measured the effects of resveratrol in combination with r-tPA in IS. Patients were divided into two groups, r-tPA^+^ placebo and r-tPA^+^ 2.5 mg/kg resveratrol (maximum 250 mg). They concluded that resveratrol could extend the narrow therapeutic time window of r-tPA. They found that r-tPA^+^ resveratrol inhibits the effects of MMP-2 and MMP-9 and improves BBB function.

Fodor et al. aimed to find whether resveratrol could serve as a secondary prophylaxis agent for the prevention of stroke [229]. They found that resveratrol supplements consumption for 12 months lowered the major risk factors associated with stroke including levels of total cholesterol, triglycerides, HDL and LDL cholesterol, basal glucose, and glycosylated hemoglobin (HbA1c).

These studies lacked measurement of long-term outcomes and to determine whether resveratrol supplement can prevent the occurrence of IS or at least decrease stroke severity and neurologic deficit degree. Future studies should consider these limitations. The outcomes of these trials are summarized in Table 3.

**Table 3:** Clinical trials with resveratrol in ischemic stroke.

Reference number	*Clinical trials*	*Type of brain injury*	*Root of resveratrol administration*	*Time of resveratrol administration*	*ResveratroL Dose*	*Evaluated parameters*	*Main findings*
[230]	Human	Patients with IS	Orally	For 30 consequent days	500 ± 10 mg/day (capsules contained 170 mg resveratrol, three capsules per day)	Systolic and diastolic blood pressures	▶ Resveratrol and placebo group did not differ significantly in Systolic and diastolic blood pressures
						NIHSS	▶ Resveratrol and placebo group did not differ significantly in NIHSS
						Barthel index	▶ Resveratrol and placebo group did not differ significantly in the Barthel index
						MRS	▶ Resveratrol and placebo group did not differ significantly in MRS
[231]	Human	Patients with IS	Orally	Simultaneously with r-tPA, with 10 % as a bolus followed by the remaining 90 % as a constant infusion over 60 min.	2.5 mg/kg	NIHSS	▶ NIHSS 0–10: IS + early r-tPA + Res > IS + delayed r-tPA + Res = IS + early r-tPA + Placebo > IS + delayed r-tPA + Placebo ▶ NIHSS 11–20: IS + early r-tPA + Res = IS + delayed r-tPA + Res = IS + early r-tPA + Placebo = IS + delayed r-tPA + Placebo ▶ NIHSS >20: IS + delayed r-tPA + Placebo > IS + early r-tPA + Res = IS + delayed r-tPA + Res = IS + early r-tPA + Placebo
						Plasma levels of MMP-2 and MMP-9	▶ Resveratrol decreased MMP-2 and MMP-9 levels: IS + delayed r-tPA + Placebo > IS + early r-tPA + Placebo > IS + delayed r-tPA + Res = IS + early r-tPA + Res
[232]	Human	Patients with IS	Orally	12-month supplementation	100 and 200 mg/patien/Day	BMI determination	▶ Resveratrol decreased BMI
						levels of total cholesterol, triglycerides, HDL and LDL cholesterol, basal glucose, and glycosylated	▶ Resveratrol decreased serum cholesterol levels ▶ Resveratrol increased HDL cholesterol levels ▶ Resveratrol decreased LDL cholesterol levels ▶ Resveratrol decreased Triglyceride levels ▶ Resveratrol decreased Glucose levels
						levels of hemoglobin (HbA1c)	▶ Resveratrol decreased HbA1c (%)

IS, Ischemic stroke.

r-tPA, Recombinant tissue plasminogen activator.

NIHSS, National Institute of Health Stroke Scale.

MRS, Modified Rankin Scale.

BMI, Body Mass Index.

HDL, High-density lipoprotein.

LDL, Low-density lipoprotein.

## 9 Conclusions

Inflammation and oxidative stress play a crucial role in stroke pathogenesis. The immune response followed by stroke triggers a cascade of events that lead to BBB dysfunction and the aggravation of neurologic deficits. Resveratrol effects multiple pathways, including SIRT1, Nrf2, and Shh which overall lead to reduced inflammation, oxidative stress, and better stroke outcomes. Resveratrol exhibits anti-inflammatory, anti-oxidative, anti-apoptotic and edema reducing effects in the stroke setting. Studies that have demonstrated resveratrol's neuroprotective role and its impact on improved stroke outcomes are mainly animal studies. Although the results of animal studies are highly generalizable to the clinical setting, future clinical trials are required to assess the potential of resveratrol in the long term and to determine whether pre-treatment with resveratrol could prevent or at least decrease stroke severity. Clinical trials have found that resveratrol reduces the factors associated with increased stroke risks such as lipid profile and blood glucose, which need further attention. Blood pressure management is a crucial matter in stroke, both in prevention and intervention. The effects of resveratrol on improving endothelial function and regulating BP seem to improve CBF. In the meantime, it seems that further studies are required to better clarify if resveratrol could serve as a therapeutic agent in the stroke setting.

## 10 Limitation

In the present study, we used all the articles in English, where the full text was available, and the subject under our investigation was based on the anti-inflammatory, anti-apoptotic, and antioxidant properties of resveratrol, including animal studies and clinical trials.

## Funding

This study was financially supported by Behbahan 10.13039/501100010301Faculty of Medical Sciences, Behbahan, Iran (Grant No: 402099).

## Data availability statement

No data was used for the research described in the article.

## CRediT authorship contribution statement

**Maryam Owjfard:** Writing – review & editing, Writing – original draft. **Zahra Rahimian:** Writing – review & editing. **Farzaneh Karimi:** Writing – review & editing. **Afshin Borhani-Haghighi:** Writing – review & editing, Visualization. **Arashk Mallahzadeh:** Writing – review & editing, Writing – original draft, Supervision.

## Declaration of competing interest

The authors declare that they have no known competing financial interests or personal relationships that could have appeared to influence the work reported in this paper.
